# Highly Evolvable: Investigating Interspecific and Intraspecific Venom Variation in Taipans (*Oxyuranus* spp.) and Brown Snakes (*Pseudonaja* spp.)

**DOI:** 10.3390/toxins15010074

**Published:** 2023-01-13

**Authors:** Jory van Thiel, Luis L. Alonso, Julien Slagboom, Nathan Dunstan, Roel M. Wouters, Cassandra M. Modahl, Freek J. Vonk, Timothy N. W. Jackson, Jeroen Kool

**Affiliations:** 1 Division of Bioanalytical Chemistry, Department of Chemistry and Pharmaceutical Sciences, Faculty of Sciences, Amsterdam Institute of Molecular and Life Sciences (AIMMS), Vrije Universiteit Amsterdam, 1081 HV Amsterdam, The Netherlands; 2 Institute of Biology Leiden, Leiden University, 2333 BE Leiden, The Netherlands; 3 Naturalis Biodiversity Center, 2333 CR Leiden, The Netherlands; 4 Centre for Analytical Sciences Amsterdam (CASA), 1012 WX Amsterdam, The Netherlands; 5 Venom Supplies Pty. Ltd., Tanunda, SA 5352, Australia; 6 Centre for Snakebite Research & Interventions, Liverpool School of Tropical Medicine, Liverpool L3 5QA, UK; 7 Australian Venom Research Unit, Department of Pharmacology and Therapeutics, University of Melbourne, Parkville, VIC 3010, Australia

**Keywords:** snake venom, venom variation, evolvability, liquid chromatography, mass spectrometry

## Abstract

Snake venoms are complex mixtures of toxins that differ on interspecific (between species) and intraspecific (within species) levels. Whether venom variation within a group of closely related species is explained by the presence, absence and/or relative abundances of venom toxins remains largely unknown. Taipans (*Oxyuranus* spp.) and brown snakes (*Pseudonaja* spp.) represent medically relevant species of snakes across the Australasian region and provide an excellent model clade for studying interspecific and intraspecific venom variation. Using liquid chromatography with ultraviolet and mass spectrometry detection, we analyzed a total of 31 venoms covering all species of this monophyletic clade, including widespread localities. Our results reveal major interspecific and intraspecific venom variation in *Oxyuranus* and *Pseudonaja* species, partially corresponding with their geographical regions and phylogenetic relationships. This extensive venom variability is generated by a combination of the absence/presence and differential abundance of venom toxins. Our study highlights that venom systems can be highly dynamical on the interspecific and intraspecific levels and underscores that the rapid toxin evolvability potentially causes major impacts on neglected tropical snakebites.

## 1. Introduction

Snake venom is an ecological functional trait that is primarily used for prey capture. Venom toxins mediate antagonistic interactions between snakes and their prey, driven by natural selection. A “chemical arms race” takes place between venomous snakes and their prey in which snake venom toxins interfere with key physiological process to capture prey, and prey animals evolve molecular resistance to counteract these harmful effects [1]. The composition and activity of venoms are co-evolving with the physiology of the prey items [2,3,4,5,6]. The utility of venom is contingent upon its evolvability, i.e., its capacity to “stay ahead” in the arms race. Indeed, it has often been conjectured that venom is a highly evolvable trait, and various mechanisms contributing to this evolvability, including multiple levels of “redundancy”, have been suggested [7,8]. Evolvability may be indexed by the capacity of a trait to accumulate variation whilst maintaining its primary function [7]. Thus, studies describing the existence of variation in venom composition and activity at various taxonomic levels (from intrapopulational to interspecific) may contribute to our understanding of venom’s evolvability. Variation may result from a myriad factors—it may be arbitrary/stochastic or selected, and the variation between snake lineages is likely to be the result of both selection acting on the ecological use of a venom toxin along with the contingent evolutionary histories of such divergent lineages [9].

Venom variation is observable on the interspecific level (between species) [7,10] and intraspecific level (within species) [11,12,13,14]. Whilst intraspecific variation is often identified between age classes (i.e., “ontogenetic shifts”) or populations (regional variation), the degree to which variation exists within populations is poorly understood. However, the existence of intrapopulational variation is predicted by the hypothesis that venom is a highly evolvable trait [15] and has been discovered by those few studies that have looked for it (e.g., Smiley-Walters et al., 2019; Rashmi et al., 2021) [16,17]. Mechanistically, the evolvability of venom is underpinned by a set of genetic processes (as reviewed by Casewell et al., 2020) [9]. Novel venom toxins can arise via protein neofunctionalization leading to functionally distinct venom components [18]. Alternative- and trans-splicing can play a role in generating venom toxin diversity [19,20]. Furthermore, not only the gain of venom toxins can drive venom variation but also the loss of venom toxin encoding genes [19,21]. Venom variation can also be facilitated by differential expression levels via gene duplications (e.g., increasing the abundance) but likely also via cis- and trans-regulatory shifts of venom toxin genes (e.g., increasing or decreasing the abundance). Furthermore, the role of stochastic gene expression may be underappreciated in generating venom variation, as well as in the origins of novel functions, including the initial “recruitment” of gene products for toxic roles within venom [22]. Thus, a complex set of molecular processes collectively influence the numbers of venom toxins and their abundance that underpin venom variation, and variation may be neutral as well as functional/selected.

A compelling model clade in which to examine the characteristics of interspecific and intraspecific venom variation is found in the medically relevant taipans (*Oxyuranus* spp.) and brown snakes (*Pseudonaja* spp.) [7,23,24,25,26,27,28,29,30,31,32]. Brown snakes are responsible for the vast majority of human snakebite envenomings in Australia [33]. In contrast, in some regions of Papua New Guinea, the coastal taipan (*Oxyuranus scutellatus*) causes most snakebite envenomings [34,35]. These genera form together the monophyletic *Oxyuranus–Pseudonaja* clade that includes a total of twelve species (excluding subspecies): three taipans and nine brown snakes. Members of this clade are widespread across the Australasian region, inhabiting a wide variety of distinct biomes. Taipans are typically mammal-eating specialists, whereas brown snakes are generalists, feeding on lizards, frogs and mammals [36,37]. The *Oxyuranus–Pseudonaja* clade is well-known for having some of the most lethally venomous snake species in the world (based on subcutaneous lethal dose 50% murine models) [38]. There are several toxins responsible for this extreme venom potency. The coagulopathic effects are caused by the prothrombinase complex, formed by factor X (catalytic subunit) and factor V (non-catalytic subunit) [7]. Furthermore, neurotoxicity is caused by presynaptic neurotoxins (e.g., phospholipase A_2_) and postsynaptic neurotoxins (e.g., three-fingers toxins) [7,24,26,39]. Some less abundant toxins include snake venom metalloproteinases, natriuretic peptides, C-type lectin-like proteins, cysteine-rich secretory proteins, Kunitz-type serine protease inhibitors, waprins and nerve growth factors [7,24,26,40].

Interspecific and intraspecific venom variation within this clade has begun to generate considerable research interest, although there is still much to be explored. [7,23,24,25,26,27,28,29,30,31,32]. In taipans, Barber et al. showed interspecific venom variation between three *Oxyuranus* species, with *O*. *temporalis* showing the least complex venom profile [23]. The relatively weak procoagulant activity of *O*. *temporalis* was explained by the low amount of procoagulant toxins in their venom [23]. In contrast, this species possessed the most potent neurotoxicity in the chick biventer cervicis nerve–muscle preparation. In addition to interspecific venom variation within the *Oxyuranus* genus, Tasoulis et al. described intraspecific venom variation in *O*. *scutellatus* using an analytical and functional assay-based approach [25]. This was performed by studying 13 venoms of individual *O*. *scutellatus* spread over four distinct localities on the north-east coast of Australia (i.e., Gladstone, Atherton Tableland, Cooktown and Saibai Island, respectively) [25]. Interestingly, they did not observe any sex-specific differences [25].

In brown snakes, Reeks et al. described interspecific venom variation within three *Pseudonaja* species (*P*. *textilis*, *P*. *nuchalis* and *P*. *aspidorhyncha*) using a proteomic and transcriptomic approach [26]. They observed variation across several major toxin families (i.e., three-finger toxins, phospholipase A_2_, Kunitz-type serine protease inhibitors and natriuretic peptides) [26]. However, the variation was most pronounced in the less abundant toxin families, for example, waprin, cystatin, and vespryn, and cobra venom factor-like proteins were only found in *P*. *textilis* venom [26]. A particularly profound example of interspecific variation (comparable to that of *O*. *temporalis*) concerns the ringed brown snake (*P*. *modesta*). Whereas the remaining eight *Pseudonaja* species show potent (though variable) in vitro coagulation venom activity, *P*. *modesta* venom completely lacks this activity [7,28,41]. Furthermore, intraspecific regional variation in *P*. *textilis* venom has long been documented between Queensland and South Australia localities [27,29,30]. The venom of Queensland specimens exhibits more potent in vitro coagulopathic and anti-plasmin activity compared to their South Australian conspecifics [29,30]. Furthermore, the mechanisms of neurotoxicity differ between the locales: they are primarily presynaptic for the Queensland specimens with greater postsynaptic neurotoxicity for the South Australian specimens [30]. Most notably, intraspecific venom variation in *Pseudonaja* species has been linked to ontogeny. The prothrombinase complex is absent in juvenile venoms resulting in a lack of procoagulant activity [7]. In contrast, this enzymatic complex is present in adult specimens resulting in potent coagulopathic activity [7]. This ontogenetic venom shift is linked to an ontogenetic dietary change in the ecological niche [7,41].

**Figure 1:**
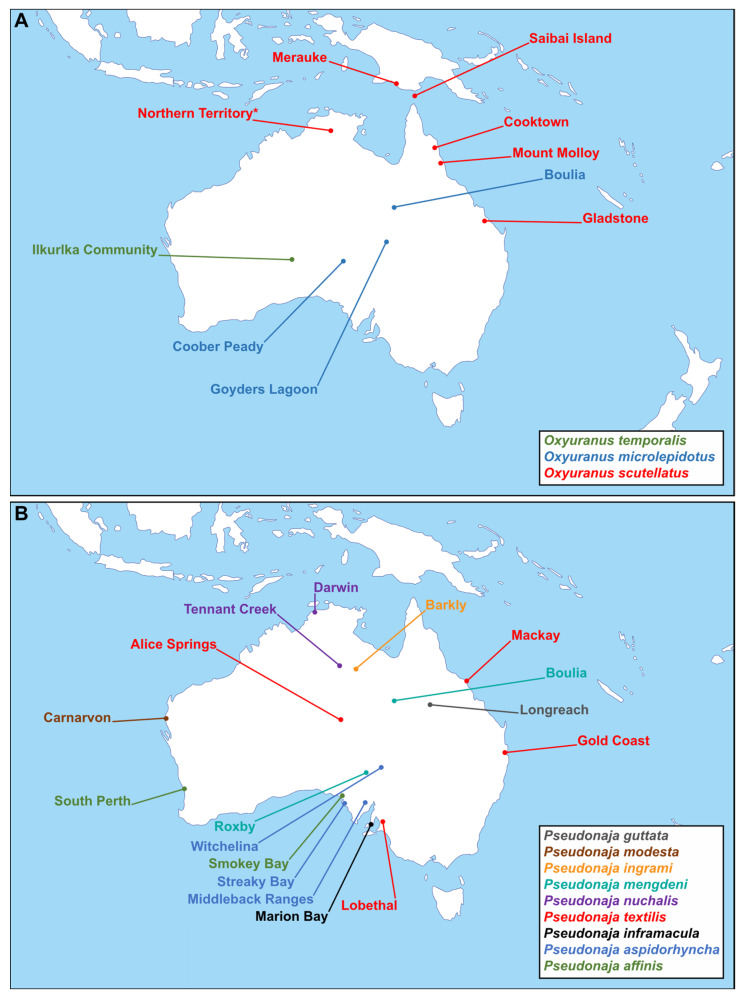
Overview of selected venom samples with their corresponding localities of origin included in this study. Each locality was designated based on the closest settlement to the capture location of each individual snake. (**A**) Schematic map of the Australasian region showing the origins of the taipan (*Oxyuranus* spp.) venom samples used in this study. The coastal taipan (*O. scutellatus*) sample originating from Northern Territory was designated to the map based on the known distribution of the species as the exact location is unknown (indicated by *). (**B**) Schematic map of the Australasian region showing the origins of the brown snake (*Pseudonaja* spp.) venom samples used in this study. Map was taken from d-maps.com (https://d-maps.com/carte.php?num_car=3322&lang=en, accessed on 21 June 2021).

In this study, we aim to further elucidate the extent of interspecific and intraspecific venom variation within the *Oxyuranus–Pseudonaja* clade using liquid chromatography with UV and mass spectrometry detection. This is the first study that applies a comprehensive and detailed analytical approach, including representative samples from all 12 species, as well as many distinct localities (Figure 1). We provide an opening of observational work that allows the testing of future research questions underpinning venom variation. The results of this study contribute to our understanding of the extent of variation in snake venoms and thus provides a window into the evolvability of this functional trait. Understanding the extent and sources of venom variation may also be important for the design and development of future treatments for human snakebite victims and may help elucidate the causes of diverging patterns of clinical sequelae following bites [9].

## 2. Results and Discussion

**Figure 2:**
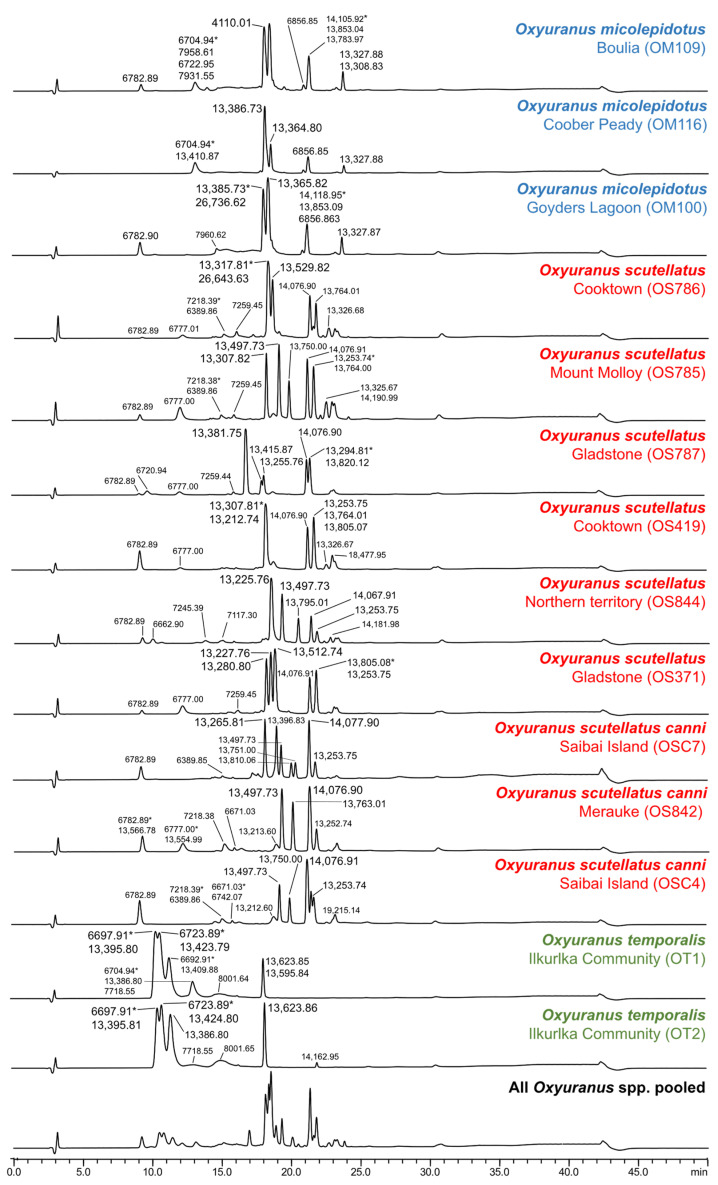
Comparative LC-UV (220 nm) measurements of taipan (*Oxyuranus* spp.) venoms. Peaks indicate the relative protein abundancy and show the corresponding masses (Da) identified using the in-parallel acquired mass spectrometry measurements. The mass with the highest intensity is indicated by an asterisk (*). The pooled sample consists of all individual taipan venoms.

**Figure 3:**
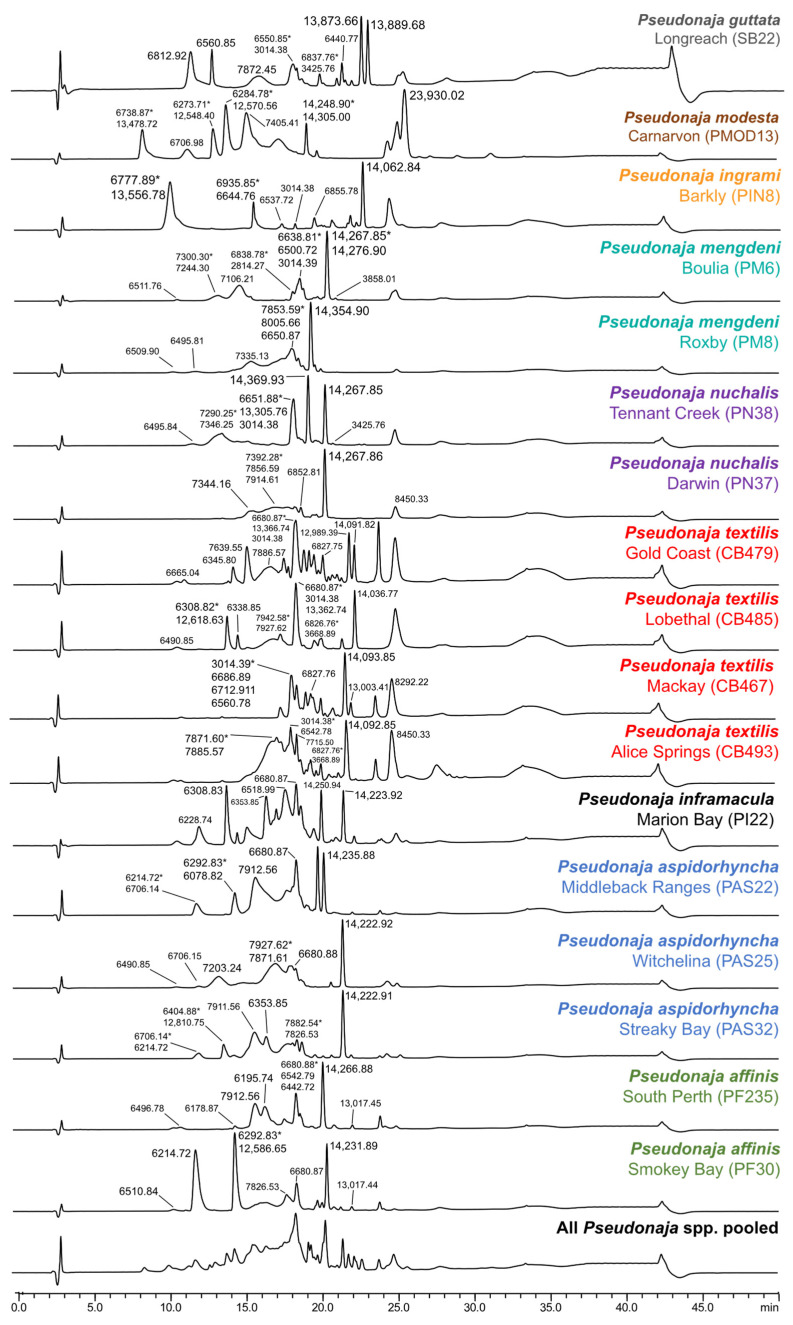
Comparative LC-UV (220 nm) measurements of brown snake (*Pseudonaja* spp.) venoms. Peaks indicate the relative protein abundance and show the corresponding masses (Da) identified using the in-parallel acquired mass spectrometry measurements. The mass with the highest MS-intensity is indicated by an asterisk (*). The pooled sample consists of all individual brown snake venoms.

We studied interspecific and intraspecific venom variation in taipans (*Oxyuranus temporalis*, *O. microlepidotus* and *O. scutellatus*) and brown snakes (*Pseudonaja guttata*, *P. modesta*, *P. ingrami*, *P. textilis*, *P. inframacula*, *P. aspidorhyncha*, *P. mengdeni*, *P. nuchalis* and *P. affinis*) using LC with UV and MS detection. Our comparative LC-UV data reveal extensive variation in taipan (*Oxyuranus* spp.) and brown snake (*Pseudonaja* spp.) venoms (Figure 2 and Figure 3). Our analysis not only indicated venom variation between the different species but also differences between the localities within a single species. The LC-UV venom profiles of all *Oxyuranus* species reveal the coastal taipan (*O. scutellatus*) as the most complex (based on the number of peaks). In contrast, the western desert taipan (*O. temporalis*) shows a less complex profile in both LC-UV and LC-MS chromatograms in terms of the number of peaks (Figure 2 and Supplementary Figures S1 and S2). This is consistent with previous findings of comparative LC measurements among all taipan species [23]. Notably, intraspecific venom variation associated with distinct localities was observed in inland taipans (*O*. *microlepidotus*, i.e., Boulia (QLD), Coober Peady (SA) and Goyders Lagoon (SA); N = 3) and coastal taipans (*O. scutellatus*, i.e., Cooktown (QLD), Mount Molloy (QLD), Gladstone (QLD), Saibai Island (QLD), Northern Territory and Merauke; N = 9). This is the first evidence of intraspecific variation in the venom composition of *O*. *microlepidotus*, although variation in the procoagulant activity between the two populations has previously been documented [28]. In the case of *O. scutellatus*, the differences in the peak distribution and peak shape in the Cooktown, Gladstone and Saibai Island localities (N = 2 per locality) indicate variation even within populations (Figure 2).

The LC-UV venom profiles of all *Pseudonaja* species show major differences indicating interspecific venom variation (Figure 3). This varies from relatively “less-complex” venom profiles in Mengden’s brown snake (*P*. *mengdeni*) to the complex venom profiles of eastern brown snakes (*P*. *textilis*). The differences in peak distribution and peak shape also suggest intraspecific venom variation in several species (Figure 3). These include *P*. *mengdeni* (Boulia (QLD) and Roxby (SA)), the northern brown snake (*P*. *nuchalis*—Tennant Creek (NT) and Darwin (NT)), the strap-snouted brown snake (*P*. *aspidorhyncha*—Witchelina (SA), Middleback Ranges (SA) and Streaky Bay (SA)) and dugite (*P*. *affinis*—South Perth (WA) and Smokey Bay (SA)). Intraspecific venom variation was most notable in *P*. *textilis* (Mackay (QLD), Gold Coast (QLD), Lobethal (SA) and Alice Springs (NT)). The venom “profiles” of each population are unique, which is consistent with previous findings between Queensland and South Australia localities [27,29,30].

To get a better understanding of the toxin species underlying the observed variation, we analyzed the in-parallel obtained MS measurements. Abundant masses were manually extracted from each venom, comprising a total of 75 abundant masses in *Oxyuranus* venoms and 112 abundant masses in *Pseudonaja* venoms. These toxins are predominantly present in the 5–8 kDa (e.g., putative three-finger toxins and Kunitz-type serine protease inhibitors) and 12–16 kDa (putative phospholipase A_2_; Supplementary Figures S1–S31) mass ranges. These three venom gene families dominate the venom compositions of the *Oxyuranus* and *Pseudonaja* species [24,25,31,32,42]. These mass ranges were found in all venoms across this clade. Additionally, a few masses were also identified in the mass ranges of >20 and <5 kDa (Supplementary Figures S1–S31).

**Figure 4:**
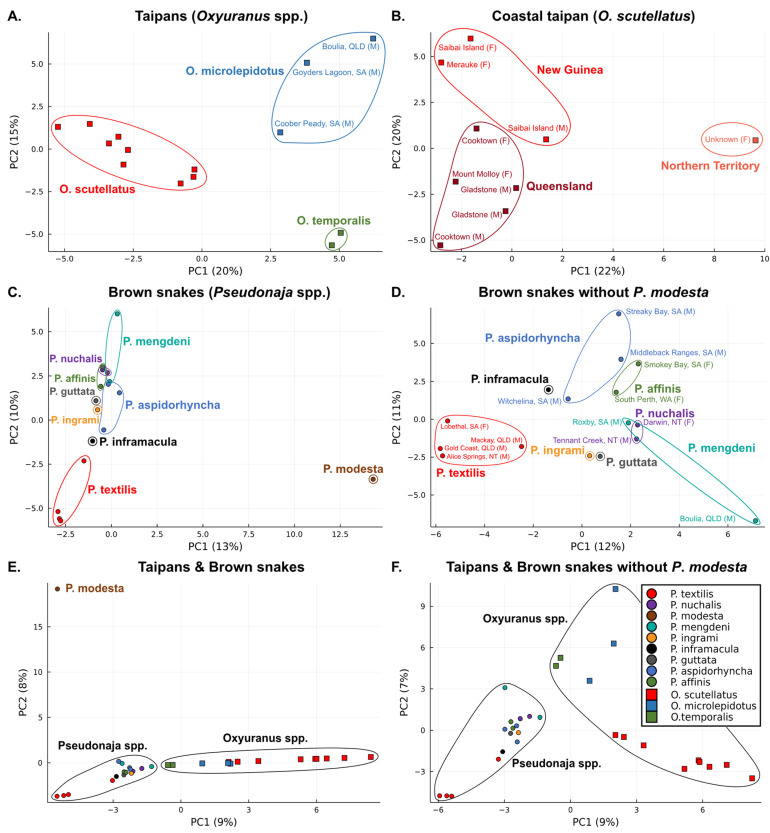
Principal component analysis (PCA) of taipan (*Oxyuranus* spp.) and brown snake (*Pseudonaja* spp.) venoms. (**A**) PCA of all taipan venoms. (**B**) PCA of coastal taipan (*O*. *scutellatus*) venoms. (**C**) PCA of all brown snake venoms. (**D**) PCA of all brown snake venom samples only excluding *P*. *modesta*. (**E**) PCA of all taipan and brown snake venoms. (**F**) PCA of all taipan and brown snake venoms only excluding *P*. *modesta*.

To visualize the relative interspecific and intraspecific venom variation, we performed principal component analyses (PCA). Our analysis of the fourteen *Oxyuranus* venoms revealed strong clustering at the species level underpinned by clear differentiation between the three species (Figure 4A). Venom variation observed within *O. microlepidotus* was associated with geography (N = 3), with Coober Peady being an isolated, distinct region compared to the Boulia and Goyders Lagoon populations (Figure 4A). This intraspecific variation is consistent with prior results demonstrating the different neutralizing potency of taipan antivenom and differences in the phospholipid-dependency of procoagulant activity between venoms from Coober Peady and Boulia populations [28]. The PCA of *O*. *scutellatus* venoms revealed major intraspecific variations that generally clustered according to geographic regions (i.e., New Guinea, Queensland and Northern Territory) (Figure 4B). However, this relative variation seems to be ambiguous between the New Guinea and Queensland regions, compared to the isolated Northern Territory populations. Intrapopulation venom variation is suggested in the Cooktown and Sabai Island localities as the venoms (N = 2 per population) from each locality did not cluster together. Venom variation on the intrapopulational level is rarely studied but has been observed in a few snake species [16,17], including *O*. *scutellatus* [25]. In contrast to the Cooktown and Sabai Island localities, the Gladstone venoms (N = 2) did cluster together. Notably, however, whilst both Gladstone samples were collected from male snakes, the Cooktown and Sabai Island venoms were collected from one male and one female specimen per locale. It is therefore possible that the observed intrapopulational variation may reflect a distinction between the venoms of male and female snakes (Figure 4B). However, as sexual variation in *O*. *scutellatus* venom was not observed in a previous study [25], this possibility requires further investigation. Venom from the Northern Territory was the most divergent (N = 1; Figure 4B).

The PCA of seventeen *Pseudonaja* venoms revealed a clear divergence between the ringed brown snake (*P*. *modesta*) and its congeners (Figure 4C). This conforms with prior studies indicating that *P*. *modesta* has a distinctive venom, being the only member of the genus lacking the procoagulant venom phenotype [7,28,41]. To get a more precise representation of venom variation among the other eight species of *Pseudonaja*, we performed an additional PCA excluding *P*. *modesta* (Figure 4D). This analysis revealed a separation between two phylogenetic clades within the *Pseudonaja*: although *P. textilis* was something of an outlier, the clade consisting of that species along with *P. inframacula*, *P. aspidorhyncha* and *P. affinis* clustered together, whereas the clade containing *P. guttata*, *P. ingrami*, *P. mengdeni* and *P. nuchalis* clustered separately [43,44]. The *P. textilis* venoms were outliers regardless of their geographic origin (Figure 4C,D). The variation in *P. aspidorhyncha*, on the other hand, was clearly patterned according to location, evidenced by the relative similarity of the samples from the Middleback Ranges and Streaky Bay, compared to Witchelina (Figure 4C,D). Interestingly, certain samples (e.g., *P. mengdeni* and *P. nuchalis*) were more similar to those of distinct species than to conspecifics, indicating intriguing patterns of intraspecific venom variation.

Finally, we performed PCA including all 31 venoms covering the complete monophyletic *Oxyuranus–Pseudonaja* clade. These analyses reveal a distinction between *Oxyuranus* and *Pseudonaja* venoms (Figure 4E). However, there is not an extreme divergence as expected given their shared common ancestor, as they make up a monophyletic clade. Furthermore, their venom compositions are also dominated by the same three venom toxin families (i.e., three finger toxins, phospholipase A_2_ and Kunitz-type serine protease inhibitors) [24,25,31,32,42]. Almost all members of the clade were relatively clustered in the proximity of each other, with the only exception being *P. modesta*, which appears to not only be an outlier amongst the brown snakes, but relative to the whole *Oxyuranus–Pseudonaja* clade itself (Figure 4E). One of the characteristics of the venoms of snakes within this clade is potent procoagulant activity, which *P. modesta* lacks [45]. Whilst this, in itself, might be enough to make *P. modesta* an outlier, it is noteworthy that *O. temporalis*, the only other member of the clade to lack the procoagulant prothrombinase toxin complex [28], was located within the taipan cluster, albeit in a position between the other two species of taipans and the brown snakes (Figure 4E). Whilst neurotoxicity is another ubiquitous feature within this clade, *O. temporalis* has the most potently neurotoxic venom amongst the taipans [23]. The lack of convergence between *P. modesta* and *O. temporalis* in the PCA analyses suggests that the latter has a relatively typical venom for a member of this clade, merely lacking the prothrombinase, whereas the venom of the former appears unique even when taking this shared absence into account (Figure 4E). This is a particularly interesting result given that the venom of both species is apparently dominated by post-synaptic three-finger toxins [7,32,46].

The divergence in venom composition may be partially explained by diet and other aspects of feeding ecology that differ between the two genera. The *Oxyuranus* species are typically mammal-eating specialists, whereas the *Pseudonaja* species are generalists, feeding on lizards and frogs as well as mammals. The relative predominance of each prey group in the diet of brown snakes differs between species, age classes and presumably seasonally according to fluctuations in prey abundance. Interestingly, juveniles brown snakes are lizard specialists, which transition (to varying degrees depending upon species) to a generalist diet with an increasing predominance of mammalian prey (especially in *P. textilis*) [36,47]. This ontogenetic dietary shift is linked to a shift in venom composition from predominantly neurotoxins (i.e., three-finger toxins) in juveniles to a highly potent procoagulant venom in adults (an activity attributable to the presence of the prothrombinase complex in the venom of adults) [7,41]. The only exception is seen in *P*. *modesta,* which feeds almost exclusively on lizards throughout its life [36]. Thus, the fact that *P. modesta* venom is the outlier within the clade may result from this unique feeding ecology selecting for a neotenic phenotype dominated by neurotoxins even in adulthood (Figure 4E). Performing PCA by excluding *P*. *modesta*, we still observed a clear divergence between both genera (Figure 4F). Brown snakes and taipans differ from one another in other aspects of feeding ecology, including prey-handling behavior.

**Figure 5:**
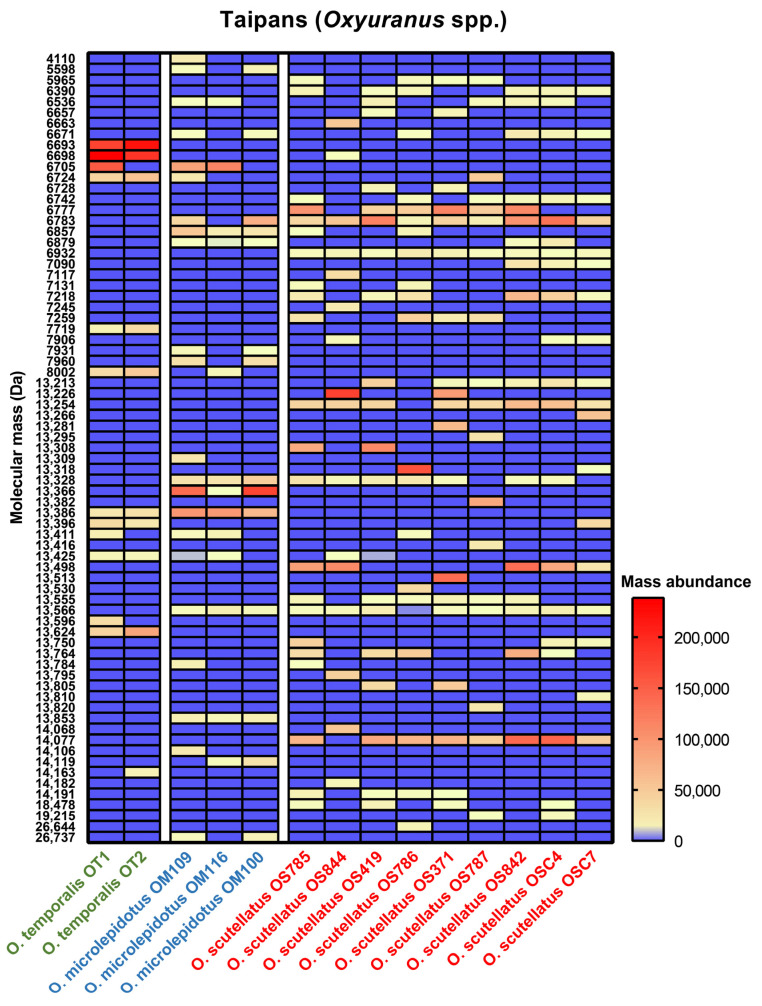
Heatmap visualization of comparative protein abundances across taipan (*Oxyuranus* spp.) venoms. Red and yellow indicate the presence of a mass, and blue indicates the absence of a mass. The mass abundance is based on the measured intensity in each venom. Localities: OT1 and OT2, Ilkurlka Community (WA); OM109, Boulia (QLD); OM116, Coober Peady (SA); OM100, Goyders Lagoon (SA); OS785, Mount Molloy (QLD); OS844, Northern Territory; OS419 and OS786, Cooktown (QLD); OS371 and OS787, Gladstone (QLD); OS842, Merauke (NG); OSC4 and OSC7, Saibai Island (NG). Key: Da, Dalton; NG, New Guinea; QLD, Queensland; SA, South Australia; WA, Western Australia.

Subsequently, we determined whether the observed variation is explained by shifts in the presence, absence and/or relative abundances of venom toxin classes. High-abundance toxins among all our *Oxyuranus* venoms, included 1 isoform <5 kDa, 29 isoforms in the 5–9 kDa range, 39 isoforms in the 13–15 kDa range and 4 isoforms >15 kDa (Figure 5). The majority of venom toxins were between 5 and 9 kDa (putative three-finger toxins and Kunitz-type serine protease inhibitors) and 13 and 15 kDa (putative phospholipase A_2_) in molecular weight. These three venom toxin families are known to make up the majority of *Oxyuranus* venoms [24,32]. We observed several species-specific masses, including 6693 Da in *O*. *temporalis*, 13,853 and 13,366 Da in *O*. *microlepidotus*, and 6932 and 14,007 Da in *O*. *scutellatus* (Figure 5). Furthermore, 13,386 Da was present in *O*. *temporalis* and *O*. *microlepidotus* but absent in all *O*. *scutellatus* venoms (Figure 5). In contrast, 6783, 13,328 and 13,566 Da were present in the majority of *O*. *microlepidotus* and *O*. *scutellatus* venoms but not in *O*. *temporalis* (Figure 5). No masses were present in both *O*. *temporalis* and *O*. *scutellatus* but absent in *O*. *microlepidotus* (Figure 5). The mass of 13,411 Da was observed in all three *Oxyuranus* species but not every sample from each species. We also observed some unique masses which were only found in a specific venom, such as 14,163 Da in *O*. *temporalis* (OT2), 13,309 Da in *O*. *microlepidotus* (OM109) and 26,444 Da in *O*. *scutellatus* (OS786) (Figure 5). In addition to the dynamic absence/presence of masses, we also observed notable differences in abundancy. For example, whilst 13,366 Da was present in all *O*. *microlepidotus* localities, its abundance varied extensively. Whereas this mass is lowly abundant in Coober Peady (OM116), its concentration increased approximately 100-fold in the samples from Boulia (OM109) and Goyders Lagoon (OM100) (Figure 5). *O*. *scutellatus* is a widespread species that is known for its intraspecific venom variation [23,24,25]. Common masses present in our *O*. *scutellatus* venoms were 6777, 6783, 13,254, 13,566 and 14,077 Da; however, their abundances varied considerably amongst samples (Figure 5 and Supplementary Figure S32). Interestingly, within *O*. *scutellatus*, the most unique masses were found in the Northern Territory (OS44; seven unique masses) (Figure 5 and Supplementary Figure S32). The PCA identified the Northern Territory as having the most divergent venom among *O*. *scutellatus* samples (Figure 4B).

**Figure 6:**
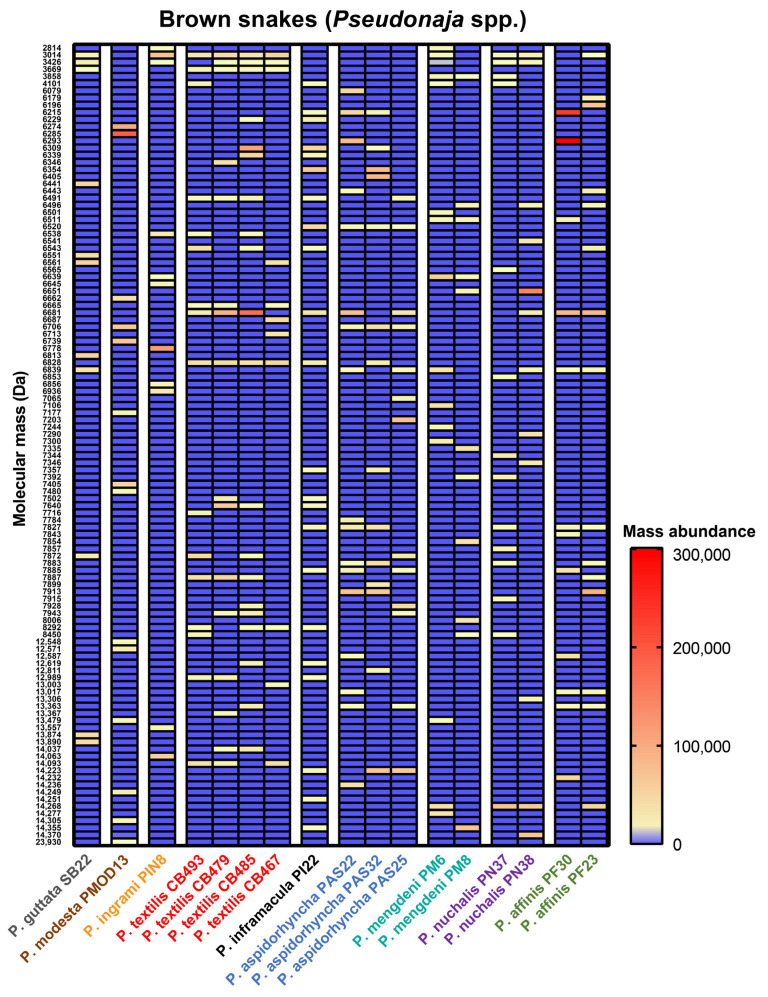
Heatmap visualization of comparative protein abundances across brown snake (*Pseudonaja* spp.) venoms. Red and yellow indicate the presence of a mass, and blue indicates the absence of a mass. The mass abundance is based on the measured intensity in each venom. Localities: SB22, Longreach (QLD); PMOD13, Carnarvon (WA); PIN8, Barkly (NT); CB493, Alice Springs (NT); CB479, Gold Coast (QLD); CB485, Lobethal (SA); CB467, Mackay (QLD); PI22, Marion Bay (SA); PAS22, Middleback Ranges (SA); PAS32, Streaky Bay (SA); PAS25, Witchelina (SA); PM6, Boulia (QLD); PM8, Roxby (SA); PN37, Darwin (NT); PN38, Tennant Creek (NT); PF30, Smokey Bay (SA); PF23, South Perth (WA). Key: Da, Dalton; QLD, Queensland; SA, South Australia; WA, Western Australia.

Amongst the highly abundant toxins recovered from our *Pseudonaja* venoms, we retrieved 6 isoforms <5 kDa, 77 isoforms between 5 and 9 kDa, 28 isoforms between 13 and 15 kDa and 1 isoform >15 kDa (Figure 6). The majority of venom toxins are found between 5 and 9 kDa (putative three-finger toxins and Kunitz-type serine protease inhibitors) and 13 and 15 kDa (putative phospholipase A_2_). These three venom toxin families are known to make up the majority of *Pseudonaja* venoms [26,31,32,42]. Whereas the greatest abundance of isoforms in *Oxyuranus* was within the 13–15 kDa range (putative phospholipase A_2_; Figure 6), in *Pseudonaja*, a greater number of isoforms were found within the 5–9 kDa range (putative three-finger toxins and Kunitz-type serine protease inhibitors; Figure 6). We did not observe any mass that was present among all *Pseudonaja* species, indicating a high degree of intraspecific variation within this genus (Figure 6). As indicated by the PCA analyses, many toxins in *P*. *modesta* are unique, providing further support for the observation that their divergence from all other members of the genus is not solely based upon the absence of the prothrombinase toxin otherwise characteristic of the venoms of adult snakes within this clade (present in all other species except *O. temporalis*).

Our data demonstrate that venom variation in *Oxyuranus* and *Pseudonaja* species is jointly shaped by toxin diversity and abundance. The prevalence of unique masses in *Pseudonaja* venoms, in contrast to those of *Oxyuranus*, suggests that brown snake venom variation is more driven by diversity of the toxin species than that of taipans. In *Oxyuranus*, fluctuations in mass abundance, i.e., shifting levels of gene expression, appear to play a more important role. In a recent study, Mason et al. described the distinct but complementary roles of toxin absence/presence and variations in expression levels in generating variation in the venom phenotypes of North American pit vipers [48]. This toxin diversity was linked to the phylogenetically distinct prey taxa in their diets [6,48]. In our case, given the degree of variation we have observed, coupled with the available data on *Pseudonaja* and *Oxyuranus*, it is not possible to tell a convincing adaptationist story that would tightly link venom variation to diverse feeding ecologies. Whilst such conjectures are useful, we should also not expect a priori that all such venom variation is directly adaptive. Rather, as venom is a highly evolvable polygenic trait, we should hypothesize the existence of a considerable degree of neutral “standing variation” both between and *within* populations [15]. This variation, generated at multiple—genetic and epigenetic—levels, is grist for the mill of adaptive selection and thus facilitates the emergence of novel functional phenotypes, without *necessarily* being functional in itself [7,49].

Further evaluation of the PCA loading plots enabled us to determine which venom protein masses constitute the principal components of each venom. The majority of venom protein masses that explain the observed variation in all PCAs are in the range of 5–8 kDa (putative three-finger toxins and Kunitz-type serine protease inhibitors) and 12–16 kDa (putative phospholipase A_2_; Supplementary Figure S33). These three venom gene families not only dominate the venom compositions of taipans and brown snakes [24,25,31,32,42] but the composition and abundance of members of these toxin classes accounts for the majority of the interspecific and intraspecific variation we observed. In addition to the fact that many variations may be neutral (see above), our descriptive data cannot be considered absolute due to the fact that the method used to assess toxin abundance is based on peak intensities in MS, which is not suitable for straightforward quantification. The abundance (i.e., ion intensities in MS) of toxin masses found are influenced by the ionization and detection sensibility of each toxin, which varies among toxins and decreases upon toxin mass. Thus, when using peak intensities, we can only assess relative abundance. Absolute venomics is able to quantify the individual toxins in venoms [50,51], but its throughput does not easily allow the analysis and data processing of large numbers of individual venom samples. Furthermore, due to the abovementioned sensitivity limitations of the MS analysis methodology used, as expected, we did not identify many proteins in the higher-mass ranges (>20 kDa) with significant abundances (Supplementary Figures S1–S31). It is well-known that higher mass proteins (e.g., snake venom metalloproteinases and the prothrombinase complex containing factor Xa and factor Va) are present in *Oxyuranus* and *Pseudonaja* venoms, and variation in the presence and/or abundance of the prothrombinase complex is a significant contributor to venom variation within the clade [7,23,24,25,26,27,40]. However, our study documents variation in the low molecular weight components of these venoms at a far greater level of detail than any previous study.

## 3. Conclusions

The present study is the first to include all members of the monophyletic *Oxyuranus–Pseudonaja* clade and reveals major interspecific and intraspecific venom variation. Using LC-UV-MS analysis, we show that interspecific and intraspecific venom variation is jointly shaped by toxin absence/presence and abundance. Venom variation associated with different geographic regions has previously been shown in *Oxyuranus* and *Pseudonaja* species, and our data confirm and extends this observation. As we were only able to include one or two individuals per locality, we expect that our data do not account for the degree of intrapopulational variation likely present in the venoms of these snakes. Adding more individuals in future studies would allow us to deepen our understanding of intrapopulational variability and thus provide a window into the evolvability of venom. It is also possible that intrapopulational, as well as intra- and interspecific venom variability could have implications for antivenom efficacy. By increasing the number of individuals, we could also test the involvement of potential evolutionary drivers (e.g., diet, geography, gender) in the evolution of the observed variation. The *Oxyuranus–Pseudonaja* clade is emerging as a promising model system for studying the molecular, evolutionary and ecological processes underlying venom variation. By utilizing such models to uncover the sources of variation in venom composition and activity, we will deepen our understanding of the evolvability of this fascinating functional trait. Such knowledge may also be applicable to the development of novel, broad-spectrum snakebite treatments.

## 4. Materials and Methods

### 4.1. Venom Preparation

Lyophilized venom samples were provided by Nathan Dunstan (Venom Supplies Pty. Ltd., Tanunda, SA, Australia). A detailed overview of venom samples that were included in this study is shown in Supplementary Table S1. The lyophilized venom samples were stored at −20 °C until reconstitution in ultrapure water (MQ) to make stock solutions of 1 mg/mL protein concentrations. MQ was obtained by purifying water using a Milli-Q Plus system (Millipore, Amsterdam, The Netherlands). Those stock solutions were then aliquoted and stored at –80 °C until further experimental work.

### 4.2. Liquid Chromatography, At-Line Nanofractionation and Mass Spectrometry

Liquid chromatography (LC) separation, followed by parallel at-line nanofractionation and mass spectrometry (MS) analysis were performed in an automated fashion. A Shimadzu HPLC system (Shimadzu, s-Hertogenbosch, The Netherlands) was used for the LC separation. The two Shimadzu LC-30AD pumps were set to a total flow rate of 500 µL/min. Next, 50 µL of venom was injected using a Shimadzu SIL-30AC autosampler. The protein concentrations of the venom samples were 2.5 mg/mL. The samples were separated with a 250 × 4.6 mm Waters Xbridge Peptide BEH300 C18 analytical column (Waters, Borehamwood, UK) with a 3.5-μm particle size and 300-Å pore size. The separations were performed in a Shimadzu CTD-30A column oven at 30 °C. Mobile phase A consisted of 98% MQ, 2% acetonitrile (ACN; Biosolve, Valkenswaard, The Netherlands) and 0.1% fluoroacetic acid (FA; Biosolve, Valkenswaard, The Netherlands), and mobile phase B consisted of 98% ACN, 2% MQ and 0.1% FA. The gradient of mobile phase B which was used for separation involved a linear increase in mobile phase B from 0 to 20% in 5 min, following an increase in solvent B from 20 to 40% B in 25 min, then a linear increase from 40 to 90% B in 4 min, followed by a 5 min isocratic elution at 90% B, and finally, the column was equilibrated for 10 min at 100% mobile phase A. The column effluent was split post-column in a 1:9 volume ratio. The smaller fraction was transferred to an ultraviolet detector (Shimadzu SPD-M30A photodiode array detector, recording UV data in the range of 200 to 300 nm), followed by a MaXis II Quadrupole time-of-flight (QTOF) mass spectrometer (Bruker Daltonics, Billerica, MA, USA). The mass spectrometer was equipped with an electrospray ionization source (ESI) which operated in positive-ion mode. The following ESI source parameters were used: (i) capillary voltage 3.5 kV, (ii) source temperature 200 °C, (iii) nebulizer at 0.8 Bar and (iv) dry gas flow 6 L/min. In addition, the following mass analyzer parameters were used: (i) mass range *m/z* 500–5500 range, (ii) in-source collision-induced dissociation (isCID) energy transfer 200 eV and (iii) 1 average spectrum was stored per second. After the post-column split, the larger amount of the eluent was directed to the waste (or when desired, transferred to a FractioMate^TM^ FRM100 nanofraction collector (Spark Holland and VU Amsterdam, Emmen and Amsterdam, The Netherlands) for fraction collection).

### 4.3. Data Analysis

The MS data were analyzed as follows: mass spectra were averaged from the TIC (total ion current)-MS chromatogram. The 10–15 most abundant venom toxins observed in the TIC were manually extracted from each MS data set by plotting the highest intensity charge state, which resulted in the extracted ion chromatograms (EICs). Deconvolution of the average mass spectra assigned charge states to the observed masses, which resulted in monoisotopic masses. The 10–15 most abundant venom toxins, with their corresponding highest intensity *m/z*-value and monoisotopic mass extracted from each venom were used to generate a database. Then, all the raw MS data for each of the analyzed venoms were screened for the charge states found for each of the 10–15 most abundant venom toxins to confirm either the presence or absence in other venom samples. These masses were considered the same if they were all within 1 Da range from each other and had the same retention time. Compass software was used for the MS data analysis (Bruker Daltonics, Billerica, MA, USA). The dataset consisting of all MS data was then subjected to principal component analysis (PCA) to visualize relative venom variation between samples. PCA was performed using an in-house script written in Julia language and supported by the scikit-learn package [52]. The LC-UV chromatograms were manually aligned for all analyzed brown snake and taipan venoms. The corresponding molecular masses were added to the LC-UV peaks. LC-UV data were extracted using the Shimadzu data analysis software (Shimadzu, s-Hertogenbosch, The Netherlands). The (i) LC-UV chromatogram, (ii) TIC chromatogram and (iii) IEC chromatogram were manually aligned for each venom. The heatmaps were constructed using GraphPad Prism 8.0 (GraphPad Software Inc., La Jolla, CA, USA).

## Data Availability

Not applicable.

## Supplementary Materials

The following supporting information can be downloaded at: https://www.mdpi.com/article/10.3390/toxins15010074/s1. The Supplementary Materials document consists of: Supplementary Table S1. Overview of the selected venom samples used in this study; Figures S1–S31. LC-UV-MS chromatogram alignments highlight the abundant venom toxins in *Oxyuranus–Pseudonaja* venoms; Figure S32. Heatmap visualization of comparative protein abundances across coastal taipan (*Oxyuranus scutellatus*) venoms. Figure S33; principal component analysis (PCA) loading scores of taipan (*Oxyuranus* spp.) and brown snake (*Pseudonaja* spp.) venoms.
