# Supplementary material for: Highly Evolvable: Investigating Interspecific and Intraspecific Venom Variation in Taipans (Oxyuranus spp.) and Brown Snakes (Pseudonaja spp.)
Source: Toxins (Basel). 2023 Jan 13;15(1):74. doi: 10.3390/toxins15010074 (PMC9864820; doi:10.3390/toxins15010074)
Supplement: Supplementary file 1 [file toxins-15-00074-s001.zip › toxins-2119825-supplementary.pdf]

Supplementary Materials

Highly evolvable: investigating interspecific and intraspecific venom variation in taipans (*Oxyuranus* spp.) and brown snakes (*Pseudonaja* spp.)

Jory van Thiel [1,2,3], Luis L. Alonso [1,4], Julien Slagboom [1,4], Nathan Dunstan [5], Roel M. Wouters [2], Cassandra M. Modahl [6], Freek J. Vonk [1,3,4], Timothy N. W. Jackson [7] and Jeroen Kool [1,4,*]

1 Division of Bioanalytical Chemistry, Department of Chemistry and Pharmaceutical Sciences, Faculty of Sciences, Amsterdam Institute of Molecular and Life Sciences (AIMMS), Vrije Universiteit Amsterdam, 1081 HV Amsterdam, The Netherlands.

2 Institute of Biology Leiden, Leiden University, 2333 BE Leiden, The Netherlands.

3 Naturalis Biodiversity Center, 2333 CR Leiden, The Netherlands

4 Centre for Analytical Sciences Amsterdam (CASA), 1012 WX Amsterdam, The Netherlands

5 Venom Supplies Pty. Ltd., Tanunda, SA 5352, Australia.

6 Centre for Snakebite Research & Interventions, Liverpool School of Tropical Medicine, Liverpool L3 5QA, UK.

7 Australian Venom Research Unit, Department of Pharmacology and Therapeutics, University of Melbourne, Parkville, VIC 3010, Australia.

Supplementary Table S1. Overview of the selected venom samples used in this study. The asterisk (*) indicates the official state of Saibai Island (Queensland, respectively). However, Saibai Island is geographically closer related to Papua New Guinea (PNG), so in our analyses we referred to it as PNG.

	Species	Gender	Sample ID	Location	State or territory
1	*Oxyuranus scutellatus*	Male	OS786	Cooktown	Queensland
2	*Oxyuranus scutellatus*	Female	OS785	Mount Molloy	Queensland
3	*Oxyuranus scutellatus*	Male	OS787	Gladstone	Queensland
4	*Oxyuranus scutellatus*	Female	OS419	Cooktown	Queensland
5	*Oxyuranus scutellatus*	Female	OS844	Unknown	Northern Territory
6	*Oxyuranus scutellatus*	Male	OS371	Gladstone	Queensland
7	*Oxyuranus scutellatus (canni)*	Male	OSC7	Saibai Island	Queensland*
8	*Oxyuranus scutellatus (canni)*	Female	OSC4	Saibai Island	Queensland*
9	*Oxyuranus scutellatus (canni)*	Female	OS842	Merauke	Merauke Regency
10	*Oxyuranus microlepidotus*	Male	OM109	Boulia	Queensland
11	*Oxyuranus microlepidotus*	Male	OM116	Coober Peady	South Australia
12	*Oxyuranus microlepidotus*	Male	OM100	Goyders Lagoon	South Australia
13	*Oxyuranus temporalis*	Male	OT1	Ilkurlka Community	Western Australia
14	*Oxyuranus temporalis*	Female	OT2	Ilkurlka Community	Western Australia
15	*Pseudonaja guttata*	Male	SB22	Longreach	Queensland
16	*Pseudonaja modesta*	Female	PMOD13	Carnarvon	Western Australia
17	*Pseudonaja ingrami*	Male	PIN8	Barkly	Northern Territory
18	*Pseudonaja mengdeni*	Male	PM6	Boulia	Queensland
19	*Pseudonaja mengdeni*	Male	PM8	Roxby	South Australia
20	*Pseudonaja nuchalis*	Male	PN38	Tennant Creek	Northern Territory
21	*Pseudonaja nuchalis*	Female	PN37	Darwin	Northern Territory
22	*Pseudonaja textilis*	Male	CB467	Mackay	Queensland
23	*Pseudonaja textilis*	Male	CB479	Gold Coast	Queensland
24	*Pseudonaja textilis*	Female	CB485	Lobethal	South Australia
25	*Pseudonaja textilis*	Male	CB493	Alice Springs	Northern Territory
26	*Pseudonaja inframacula*	Male	PI22	Marion Bay	South Australia

27	*Pseudonaja aspidorhyncha*	Male	PAS25	Witchelina	South Australia
28	*Pseudonaja aspidorhyncha*	Male	PAS22	Middleback Ranges	South Australia
29	*Pseudonaja aspidorhyncha*	Male	PAS32	Streaky Bay	South Australia
30	*Pseudonaja affinis*	Female	PF23	South Perth	Western Australia
31	*Pseudonaja affinis*	Female	PF30	Smokey Bay	South Australia

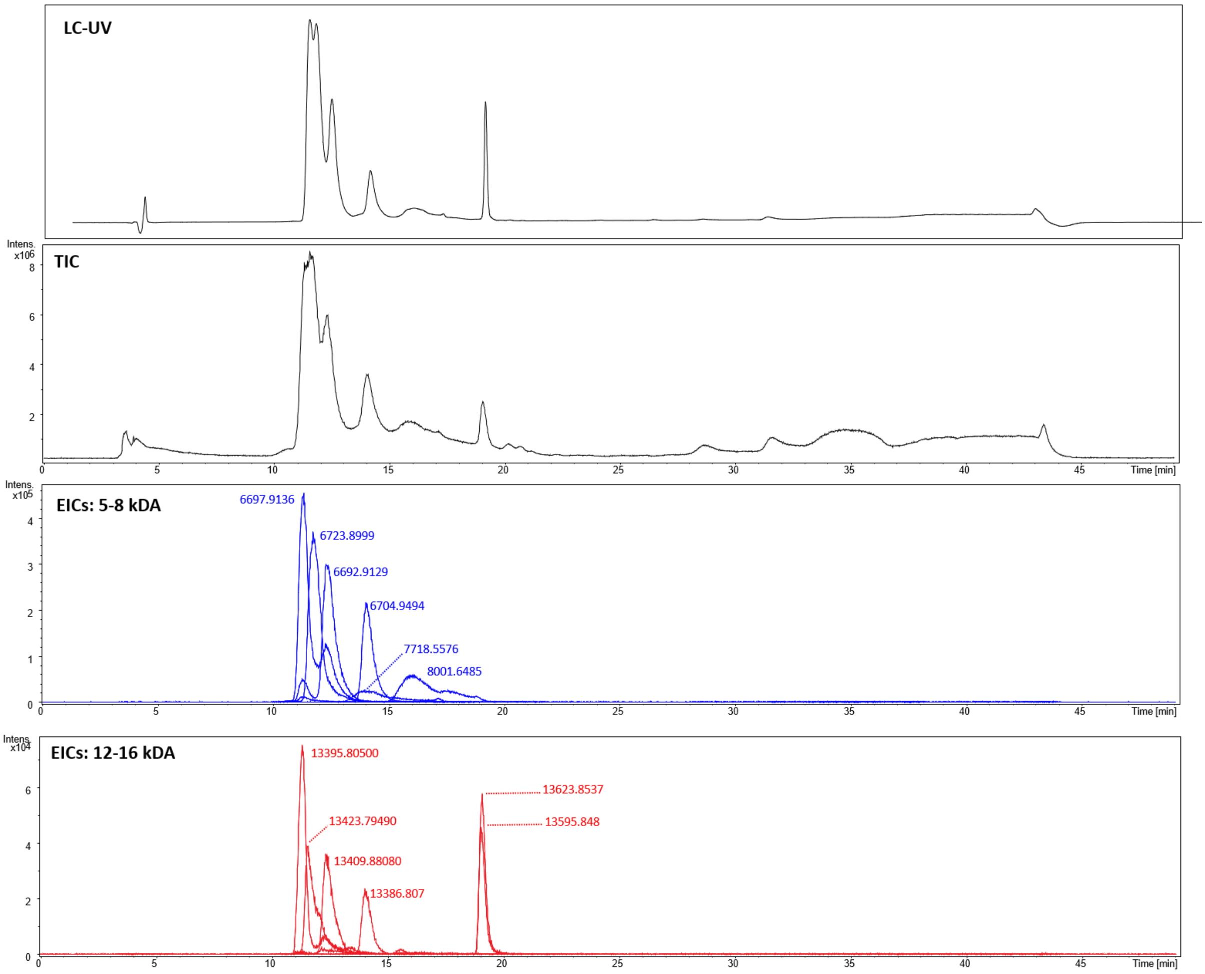

Supplementary Figure S1. LC-UV-MS chromatogram alignments highlight the abundant venom toxins in *Oxyuranus temporalis* (OT1) venom. LC-UV peaks indicate the relative protein abundancy following separation. TIC shows the summary of all measured intensities. EICs display the extracted, high-abundant masses in the venom. EICs are sorted based on their mass range to enhance visibility (i.e., mass ranges of 5-8 kDa and 12-16 kDa). Key: LC-UV, liquid chromatography coupled to ultraviolet detection; TIC, Total Ion Chromatogram; EICs, Extracted Ion Chromatograms; MS, mass spectrometry.

Oxyuranus temporalis - Ilkurlka Community (OT2)

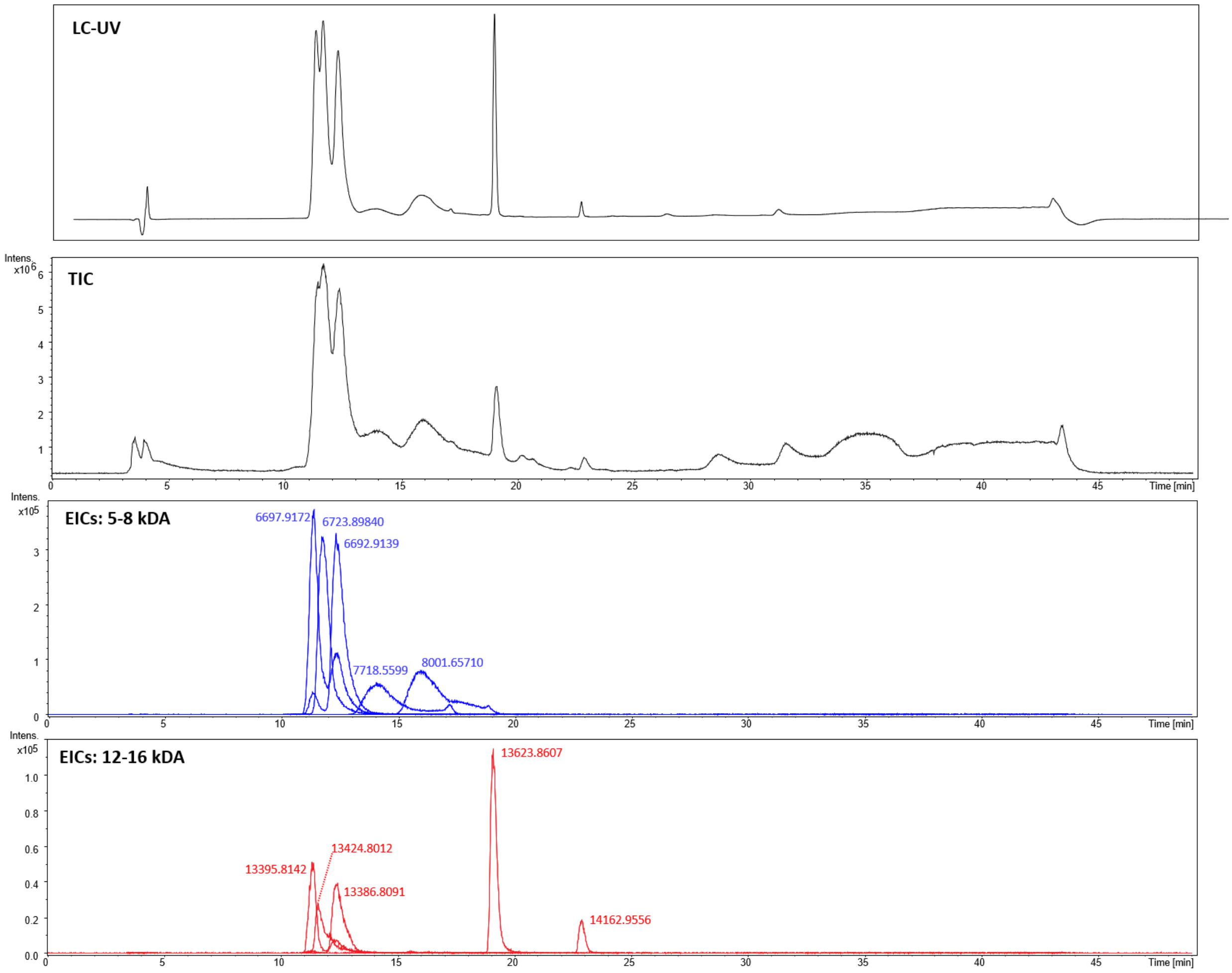

Supplementary Figure S2. LC-UV-MS chromatogram alignments highlight the abundant venom toxins in *Oxyuranus temporalis* (OT2) venom. LC-UV peaks indicate the relative protein abundancy following separation. TIC shows the summary of all measured intensities. EICs display the extracted, high-abundant masses in the venom. EICs are sorted based on their mass range to enhance visibility (i.e., mass ranges of 5-8 kDa and 12-16 kDa). Key: LC-UV, liquid chromatography coupled to ultraviolet detection; TIC, Total Ion Chromatogram; EICs, Extracted Ion Chromatograms; MS, mass spectrometry.

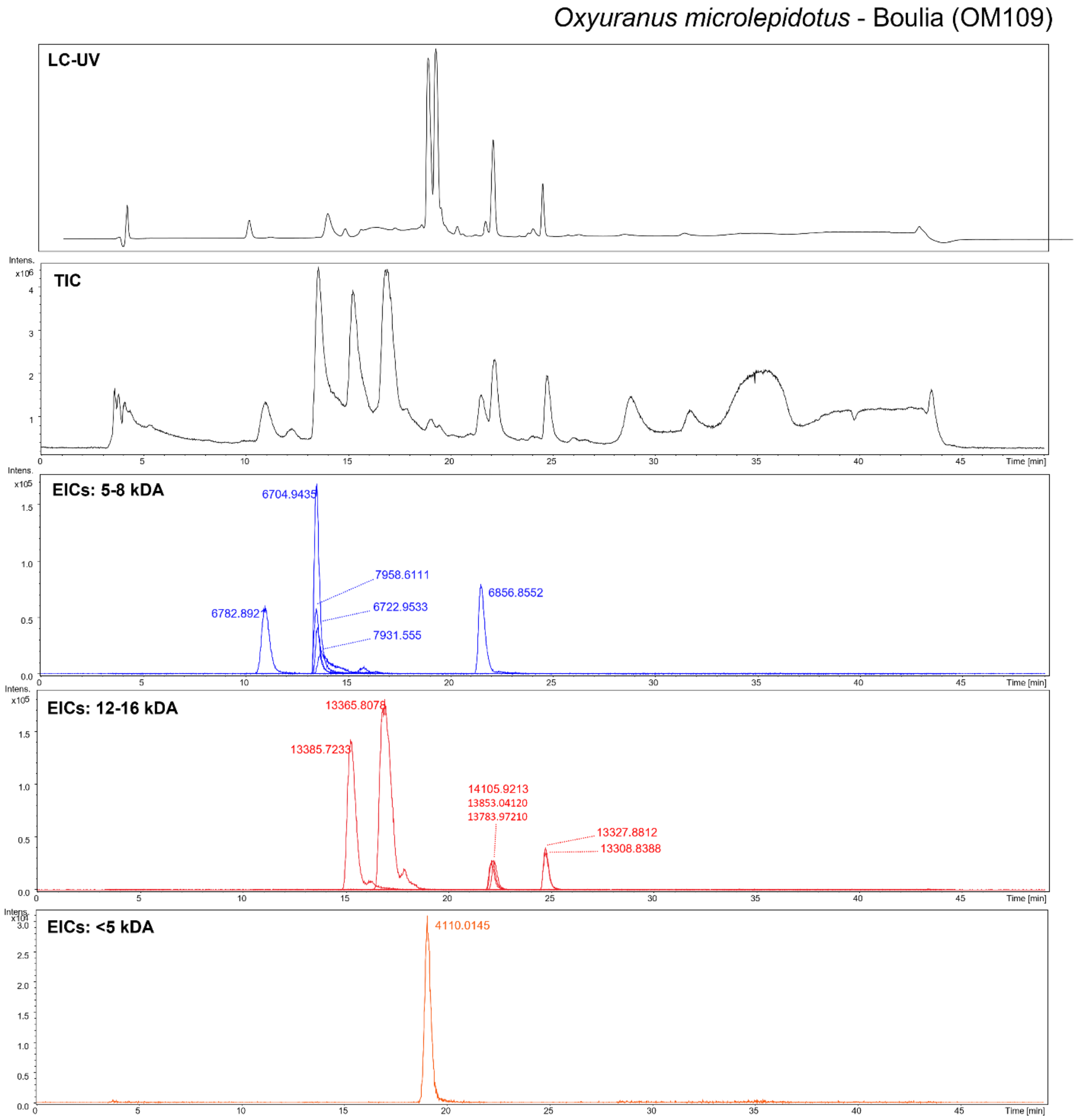

Supplementary Figure S3. LC-UV-MS chromatogram alignments highlight the abundant venom toxins in *Oxyuranus microlepidotus* (OM109) venom. LC-UV peaks indicate the relative protein abundancy following separation. TIC shows the summary of all measured intensities. EICs display the extracted, high-abundant masses in the venom. EICs are sorted based on their mass range to enhance visibility (i.e., mass ranges of 5-8 kDa and 12-16 kDa). Key: LC-UV, liquid chromatography coupled to ultraviolet detection; TIC, Total Ion Chromatogram; EICs, Extracted Ion Chromatograms; MS, mass spectrometry.

Oxyuranus microlepidotus - Coober Peady (OM116)

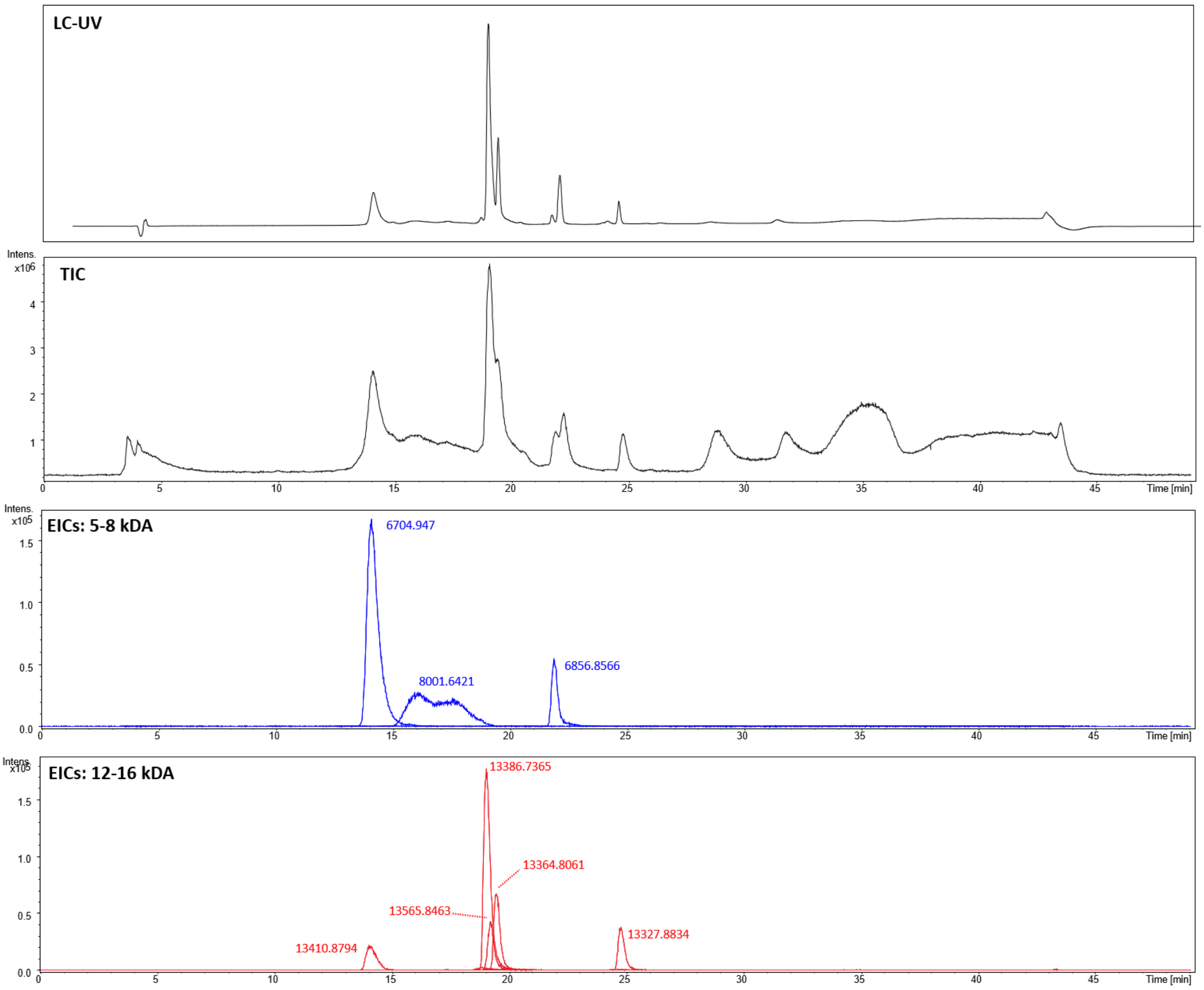

Supplementary Figure S4. LC-UV-MS chromatogram alignments highlight the abundant venom toxins in *Oxyuranus microlepidotus* (OM116) venom. LC-UV peaks indicate the relative protein abundancy following separation. TIC shows the summary of all measured intensities. EICs display the extracted, high-abundant masses in the venom. EICs are sorted based on their mass range to enhance visibility (i.e., mass ranges of 5-8 kDa and 12-16 kDa). Key: LC-UV, liquid chromatography coupled to ultraviolet detection; TIC, Total Ion Chromatogram; EICs, Extracted Ion Chromatograms; MS, mass spectrometry.

Oxyuranus microlepidotus - Goyders Lagoon (OM100)

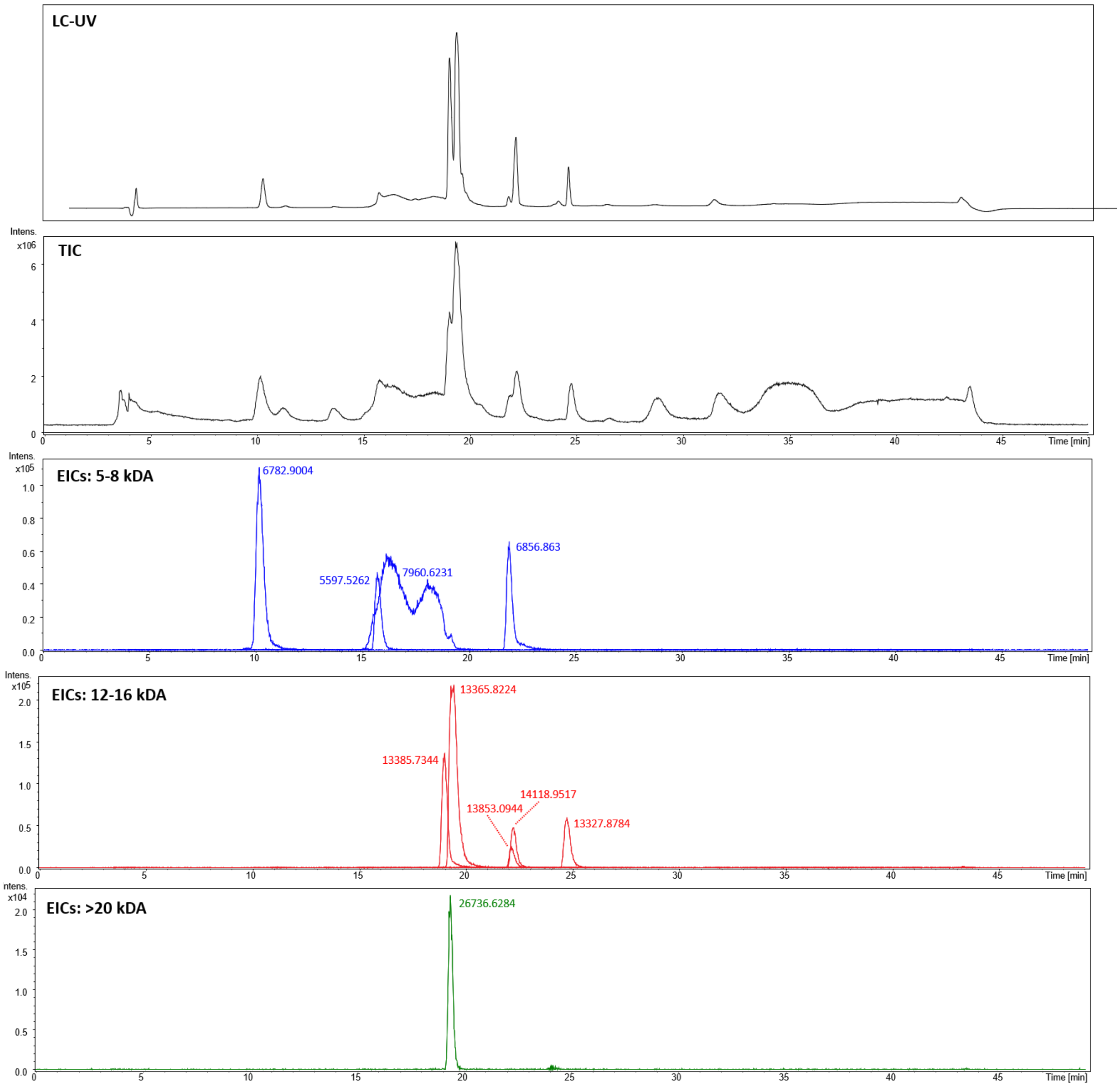

Supplementary Figure S5. LC-UV-MS chromatogram alignments highlight the abundant venom toxins in *Oxyuranus microlepidotus* (OM100) venom. LC-UV peaks indicate the relative protein abundancy following separation. TIC shows the summary of all measured intensities. EICs display the extracted, high-abundant masses in the venom. EICs are sorted based on their mass range to enhance visibility (i.e., mass ranges of 5-8 kDa, 12-16 kDa and >20 kDa). Key: LC-UV, liquid chromatography coupled to ultraviolet detection; TIC, Total Ion Chromatogram; EICs, Extracted Ion Chromatograms; MS, mass spectrometry.

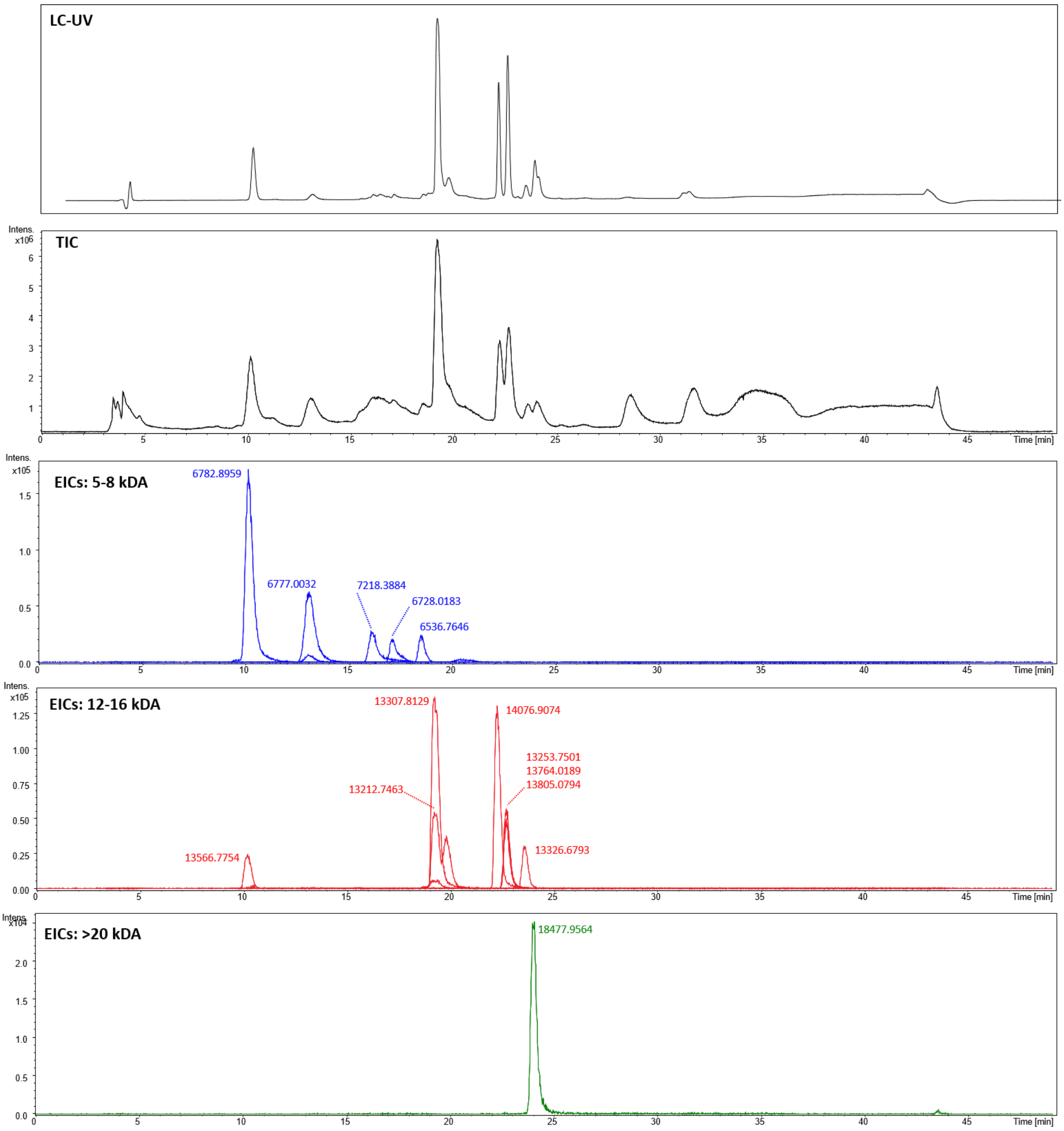

Supplementary Figure S6. LC-UV-MS chromatogram alignments highlight the abundant venom toxins in *Oxyuranus scutellatus* (OS419) venom. LC-UV peaks indicate the relative protein abundancy following separation. TIC shows the summary of all measured intensities. EICs display the extracted, high-abundant masses in the venom. EICs are sorted based on their mass range to enhance visibility (i.e., mass ranges of 5-8 kDa, 12-16 kDa and >20 kDa). Key: LC-UV, liquid chromatography coupled to ultraviolet detection; TIC, Total Ion Chromatogram; EICs, Extracted Ion Chromatograms; MS, mass spectrometry.

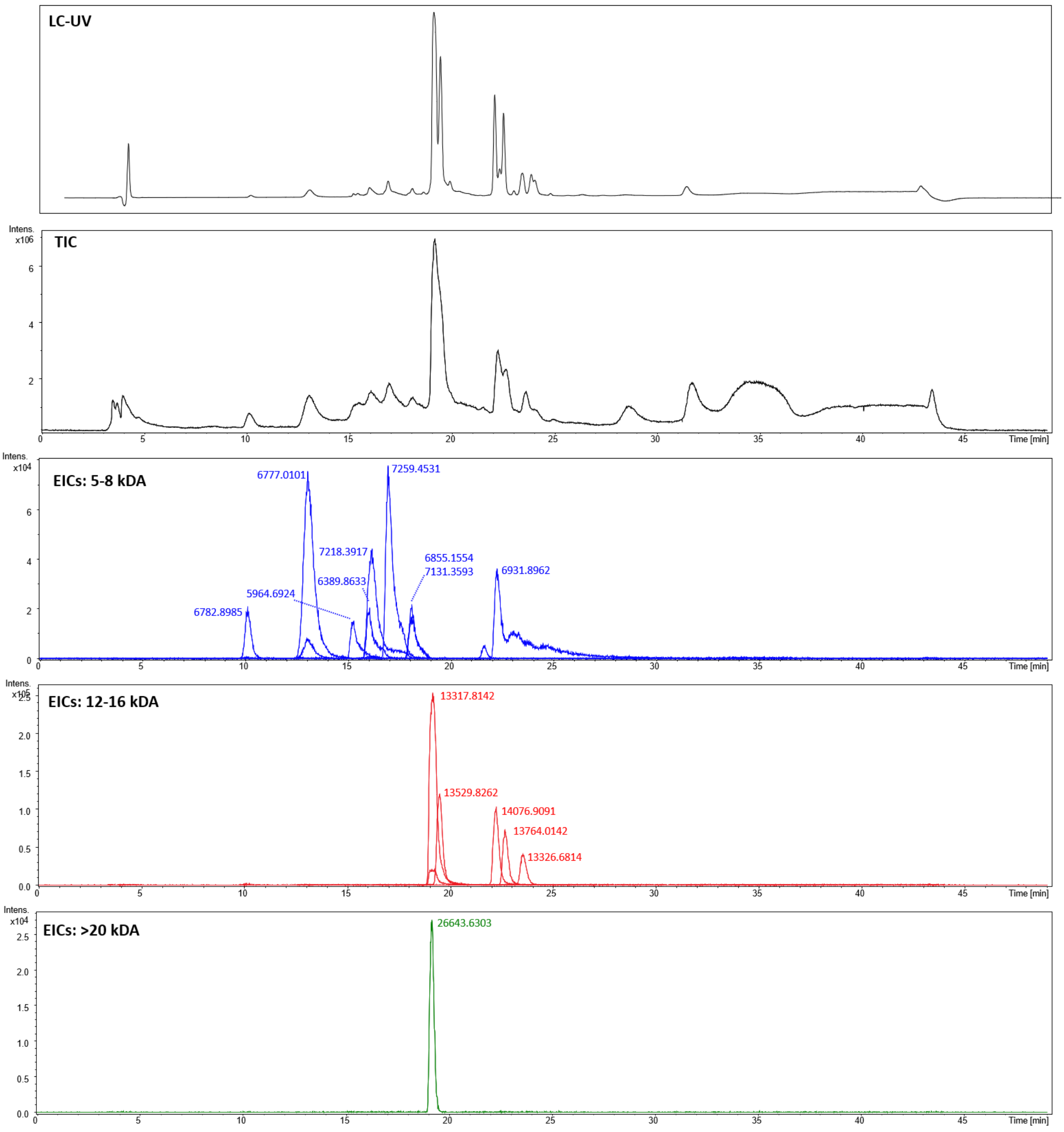

Supplementary Figure S7. LC-UV-MS chromatogram alignments highlight the abundant venom toxins in *Oxyuranus scutellatus* (OS786) venom. LC-UV peaks indicate the relative protein abundancy following separation. TIC shows the summary of all measured intensities. EICs display the extracted, high-abundant masses in the venom. EICs are sorted based on their mass range to enhance visibility (i.e., mass ranges of 5-8 kDa, 12-16 kDa and >20 kDa). Key: LC-UV, liquid chromatography coupled to ultraviolet detection; TIC, Total Ion Chromatogram; EICs, Extracted Ion Chromatograms; MS, mass spectrometry.

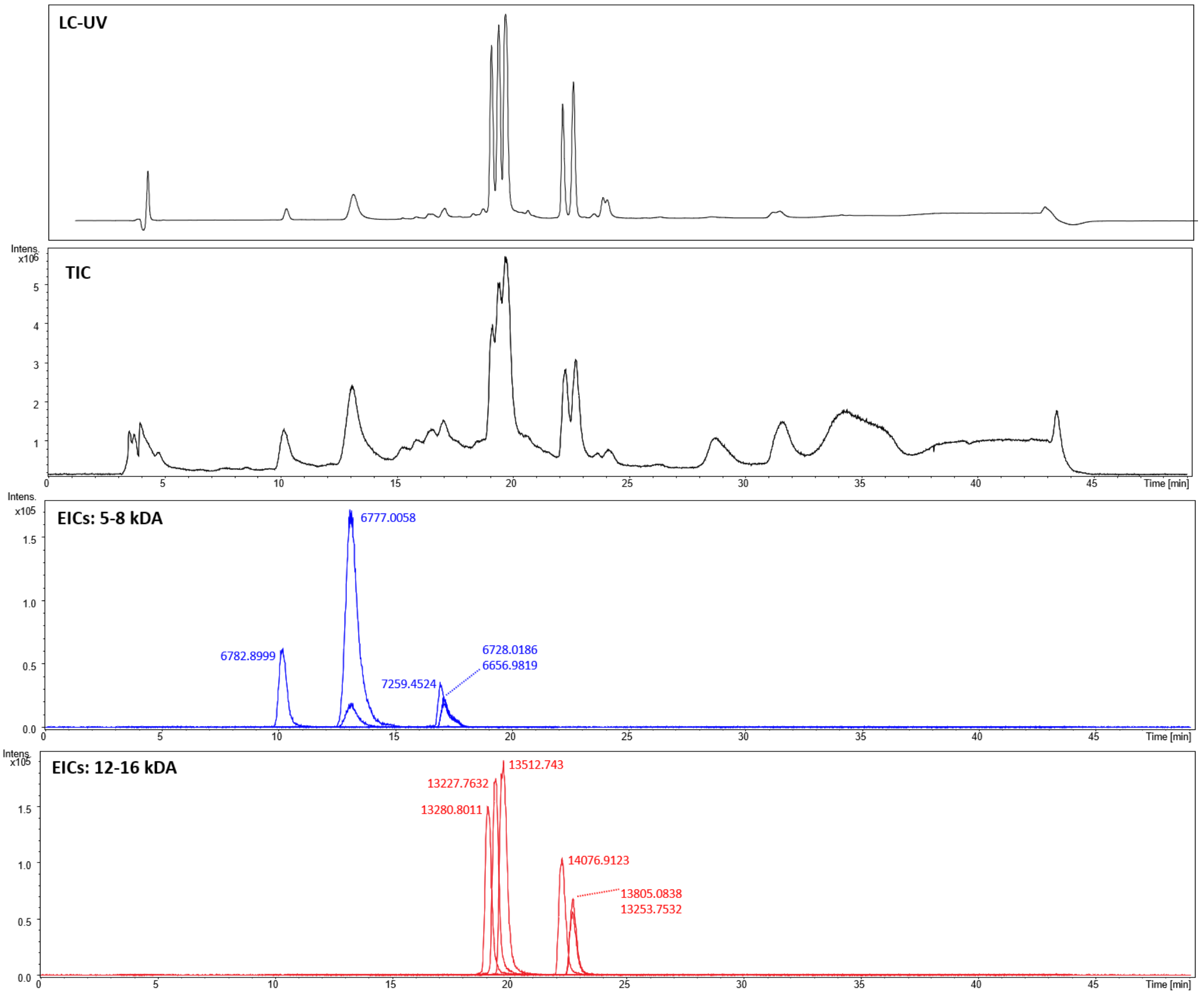

Supplementary Figure S8. LC-UV-MS chromatogram alignments highlight the abundant venom toxins in *Oxyuranus scutellatus* (OS371) venom. LC-UV peaks indicate the relative protein abundancy following separation. TIC shows the summary of all measured intensities. EICs display the extracted, high-abundant masses in the venom. EICs are sorted based on their mass range to enhance visibility (i.e., mass ranges of 5-8 kDa and 12-16 kDa). Key: LC-UV, liquid chromatography coupled to ultraviolet detection; TIC, Total Ion Chromatogram; EICs, Extracted Ion Chromatograms; MS, mass spectrometry.

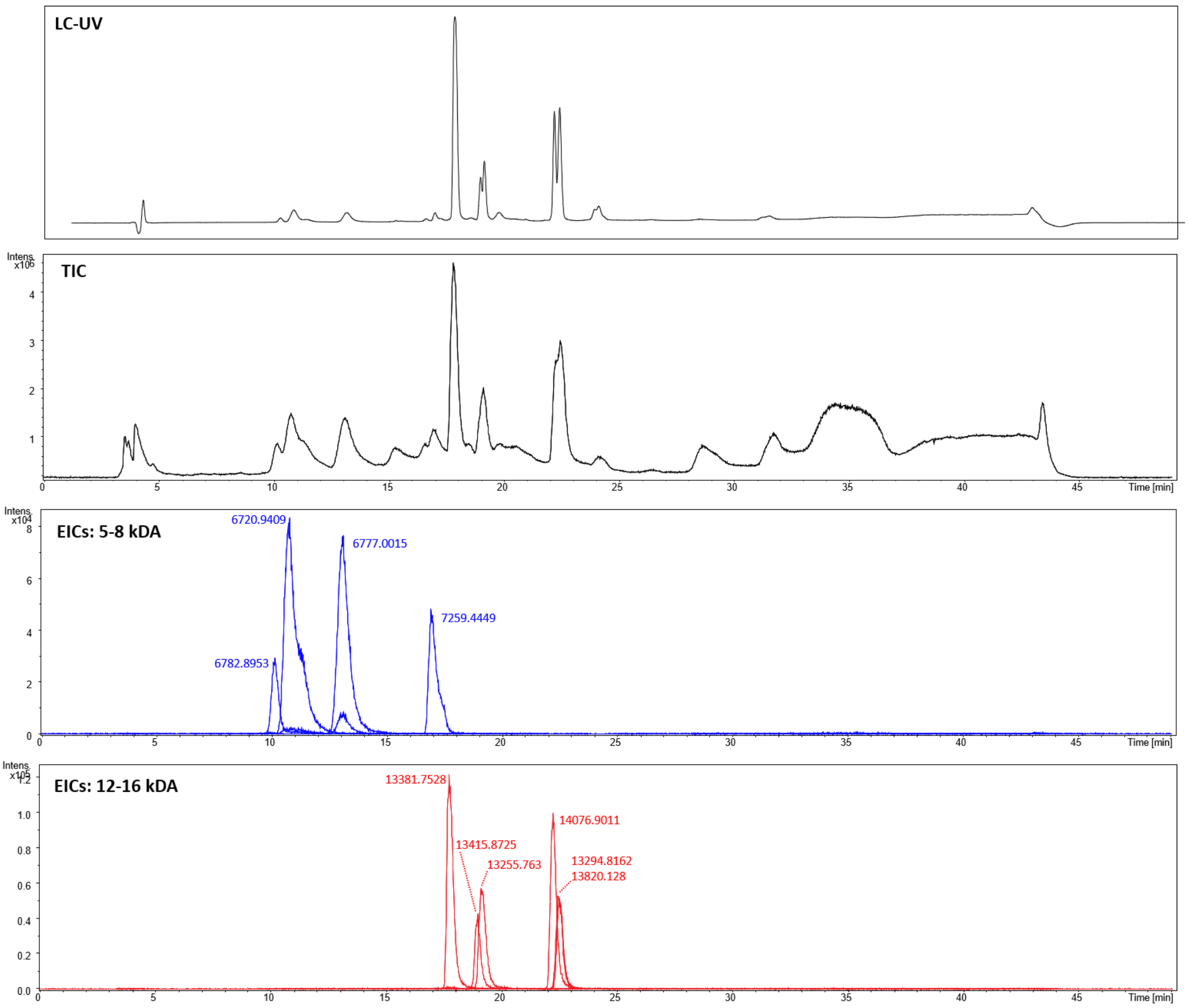

Supplementary Figure S9. LC-UV-MS chromatogram alignments highlight the abundant venom toxins in *Oxyuranus scutellatus* (OS787) venom. LC-UV peaks indicate the relative protein abundancy following separation. TIC shows the summary of all measured intensities. EICs display the extracted, high-abundant masses in the venom. EICs are sorted based on their mass range to enhance visibility (i.e., mass ranges of 5-8 kDa and 12-16 kDa). Key: LC-UV, liquid chromatography coupled to ultraviolet detection; TIC, Total Ion Chromatogram; EICs, Extracted Ion Chromatograms; MS, mass spectrometry.

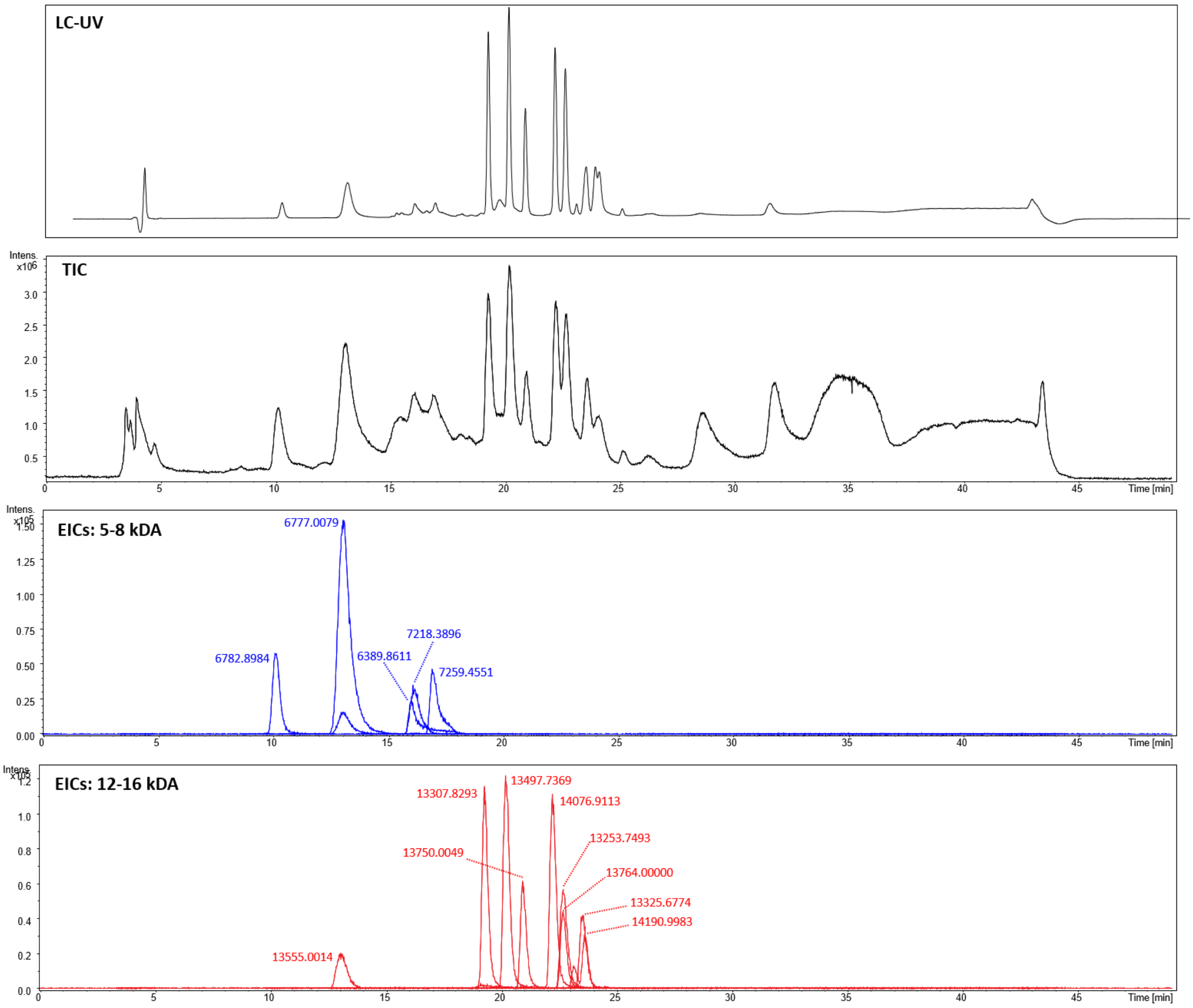

Supplementary Figure S10. LC-UV-MS chromatogram alignments highlight the abundant venom toxins in *Oxyuranus scutellatus* (OS785) venom. LC-UV peaks indicate the relative protein abundancy following separation. TIC shows the summary of all measured intensities. EICs display the extracted, high-abundant masses in the venom. EICs are sorted based on their mass range to enhance visibility (i.e., mass ranges of 5-8 kDa and 12-16 kDa). Key: LC-UV, liquid chromatography coupled to ultraviolet detection; TIC, Total Ion Chromatogram; EICs, Extracted Ion Chromatograms; MS, mass spectrometry.

Oxyuranus scutellatus – Northern Territory (OS844)

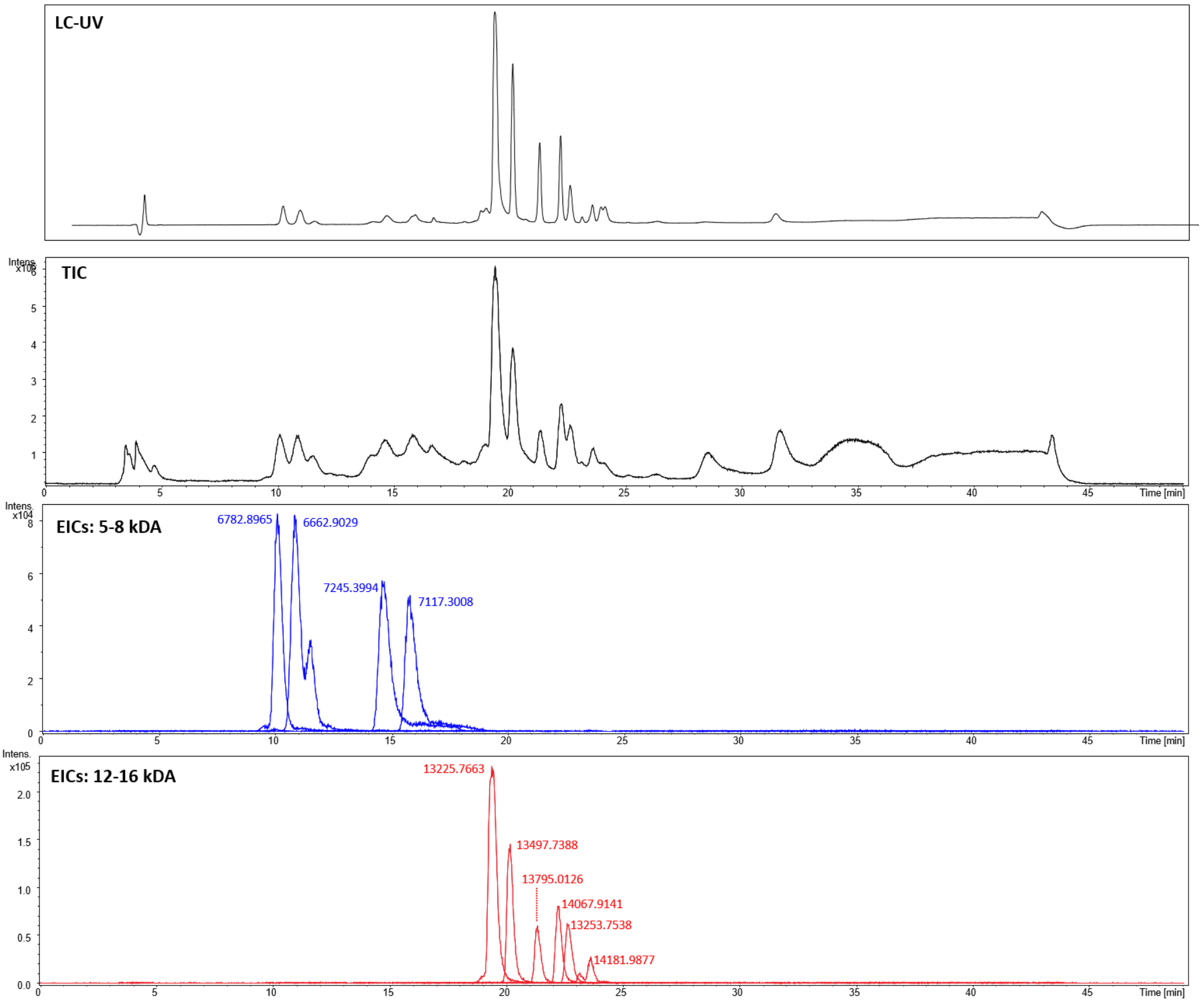

Supplementary Figure S11. LC-UV-MS chromatogram alignments highlight the abundant venom toxins in *Oxyuranus scutellatus* (OS844) venom. LC-UV peaks indicate the relative protein abundancy following separation. TIC shows the summary of all measured intensities. EICs display the extracted, high-abundant masses in the venom. EICs are sorted based on their mass range to enhance visibility (i.e., mass ranges of 5-8 kDa and 12-16 kDa). Key: LC-UV, liquid chromatography coupled to ultraviolet detection; TIC, Total Ion Chromatogram; EICs, Extracted Ion Chromatograms; MS, mass spectrometry.

Oxyuranus scutellatus canni - Merauke (OS842)

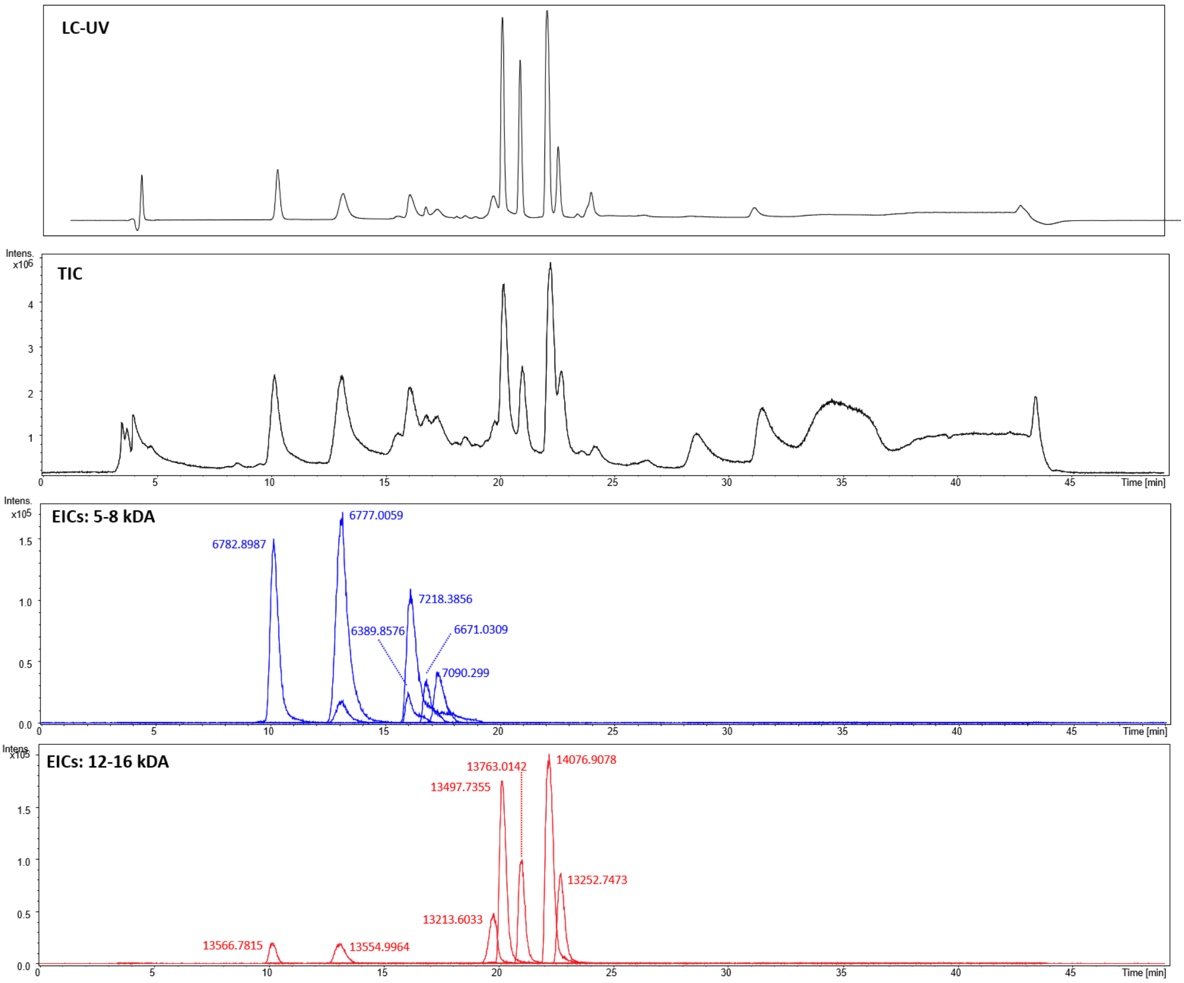

Supplementary Figure S12. LC-UV-MS chromatogram alignments highlight the abundant venom toxins in *Oxyuranus scutellatus canni* (OS842) venom. LC-UV peaks indicate the relative protein abundancy following separation. TIC shows the summary of all measured intensities. EICs display the extracted, high-abundant masses in the venom. EICs are sorted based on their mass range to enhance visibility (i.e., mass ranges of 5-8 kDa and 12-16 kDa). Key: LC-UV, liquid chromatography coupled to ultraviolet detection; TIC, Total Ion Chromatogram; EICs, Extracted Ion Chromatograms; MS, mass spectrometry.

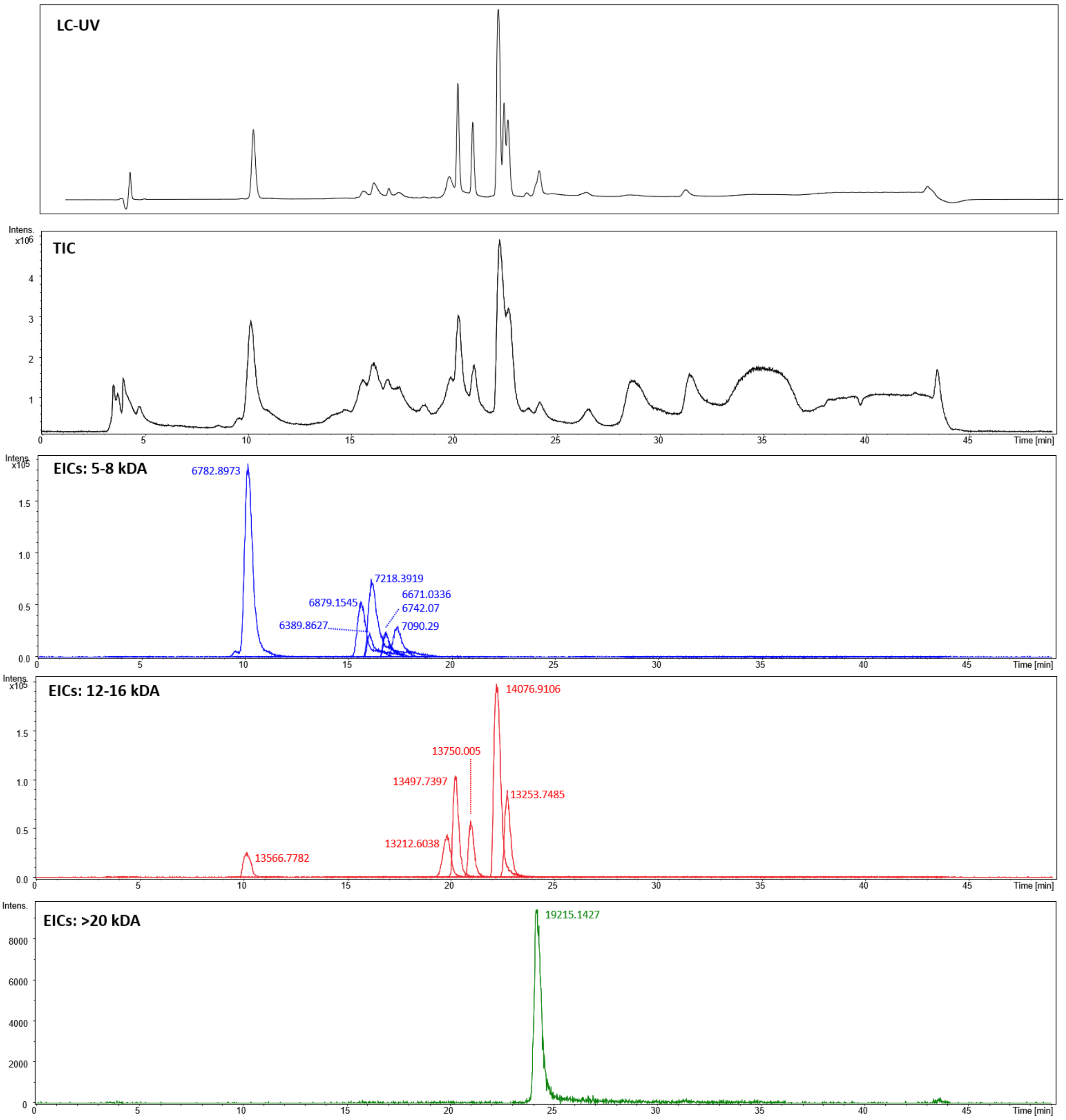

Supplementary Figure S13. LC-UV-MS chromatogram alignments highlight the abundant venom toxins in *Oxyuranus scutellatus canni* (OSC4) venom. LC-UV peaks indicate the relative protein abundancy following separation. TIC shows the summary of all measured intensities. EICs display the extracted, high-abundant masses in the venom. EICs are sorted based on their mass range to enhance visibility (i.e., mass ranges of 5-8 kDa, 12-16 kDa and >20 kDa). Key: LC-UV, liquid chromatography coupled to ultraviolet detection; TIC, Total Ion Chromatogram; EICs, Extracted Ion Chromatograms; MS, mass spectrometry.

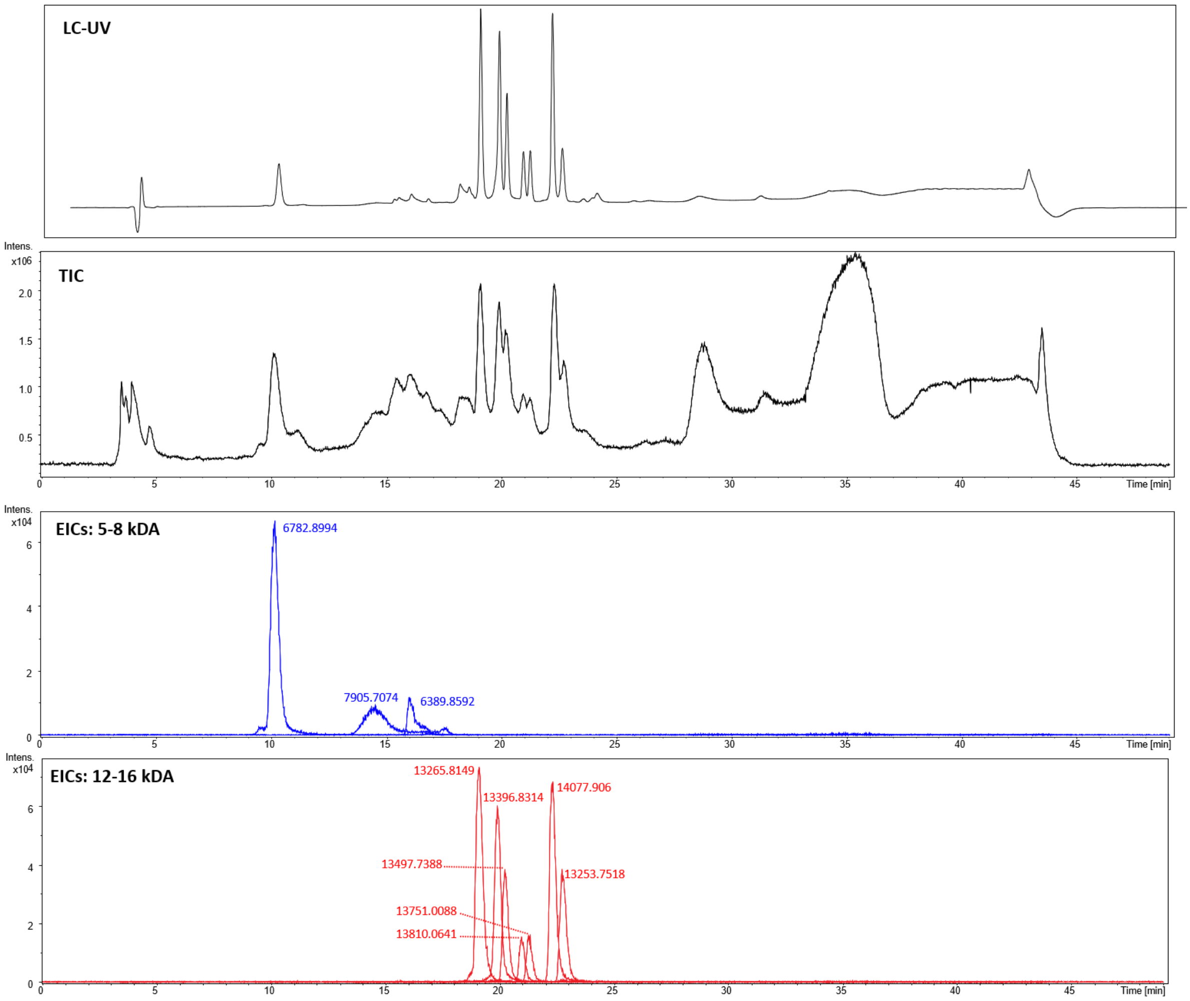

Supplementary Figure S14. LC-UV-MS chromatogram alignments highlight the abundant venom toxins in *Oxyuranus scutellatus canni* (OSC7) venom. LC-UV peaks indicate the relative protein abundancy following separation. TIC shows the summary of all measured intensities. EICs display the extracted, high-abundant masses in the venom. EICs are sorted based on their mass range to enhance visibility (i.e., mass ranges of 5-8 kDa and 12-16 kDa). Key: LC-UV, liquid chromatography coupled to ultraviolet detection; TIC, Total Ion Chromatogram; EICs, Extracted Ion Chromatograms; MS, mass spectrometry.

Pseudonaja affinis - Smokey Bay (PF30)

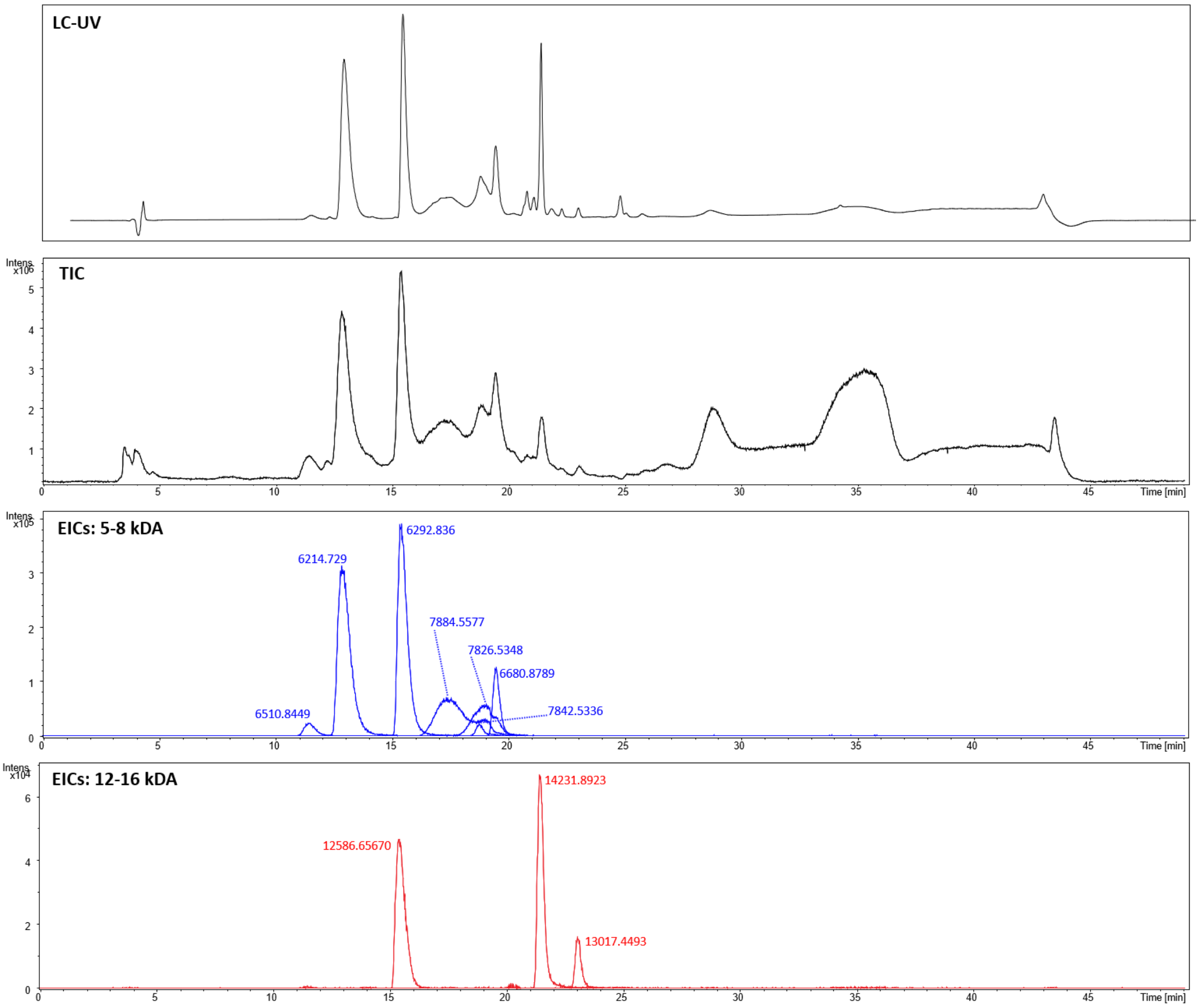

Supplementary Figure S15. LC-UV-MS chromatogram alignments highlight the abundant venom toxins in *Pseudonaja affinis* (PF30) venom. LC-UV peaks indicate the relative protein abundancy following separation. TIC shows the summary of all measured intensities. EICs display the extracted, high-abundant masses in the venom. EICs are sorted based on their mass range to enhance visibility (i.e., mass ranges of 5-8 kDa and 12-16 kDa). Key: LC-UV, liquid chromatography coupled to ultraviolet detection; TIC, Total Ion Chromatogram; EICs, Extracted Ion Chromatograms; MS, mass spectrometry.

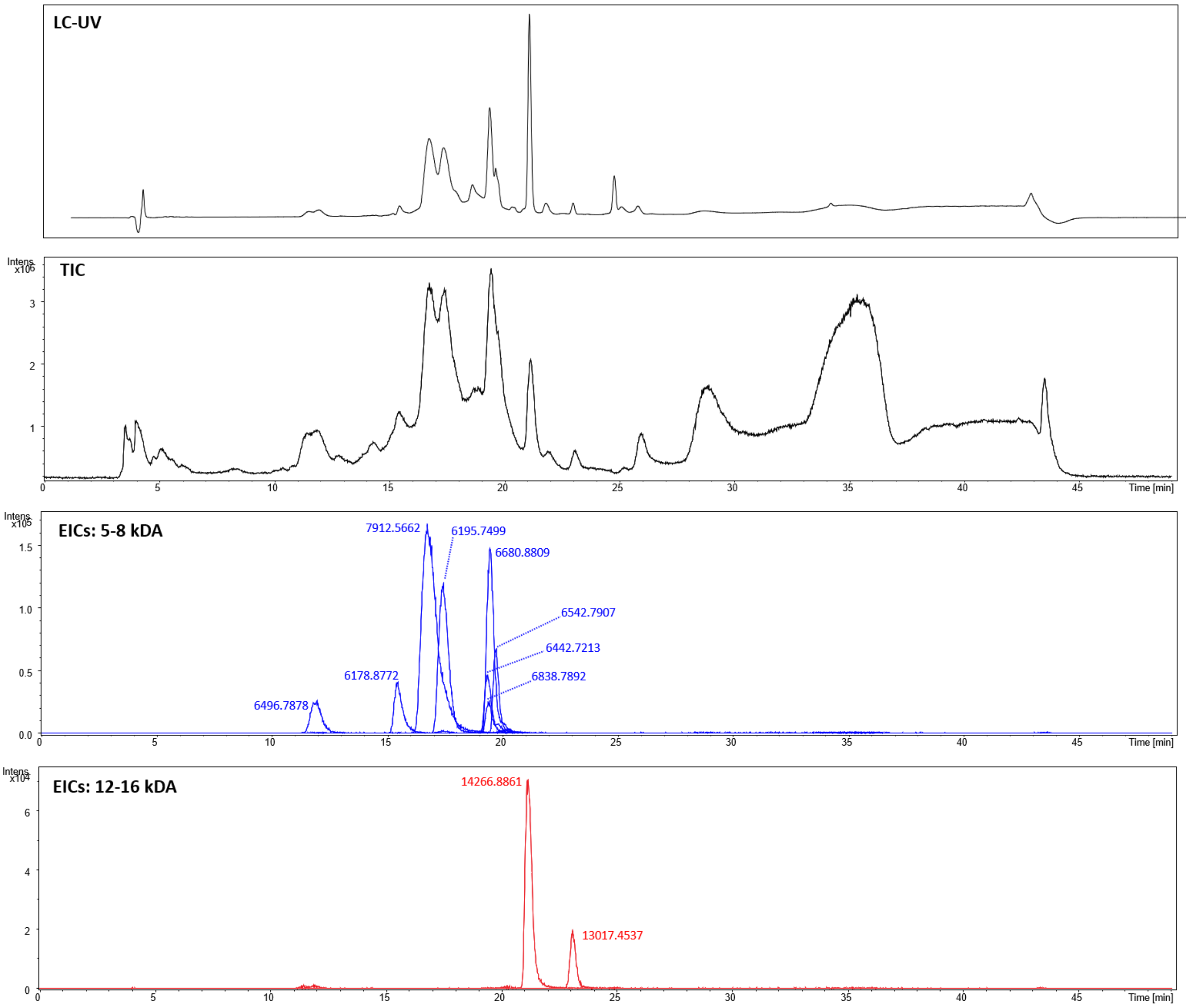

Supplementary Figure S16. LC-UV-MS chromatogram alignments highlight the abundant venom toxins in *Pseudonaja affinis* (PF23) venom. LC-UV peaks indicate the relative protein abundancy following separation. TIC shows the summary of all measured intensities. EICs display the extracted, high-abundant masses in the venom. EICs are sorted based on their mass range to enhance visibility (i.e., mass ranges of 5-8 kDa and 12-16 kDa). Key: LC-UV, liquid chromatography coupled to ultraviolet detection; TIC, Total Ion Chromatogram; EICs, Extracted Ion Chromatograms; MS, mass spectrometry.

Pseudonaja aspidorhyncha - Middleback Ranges (PAS22)

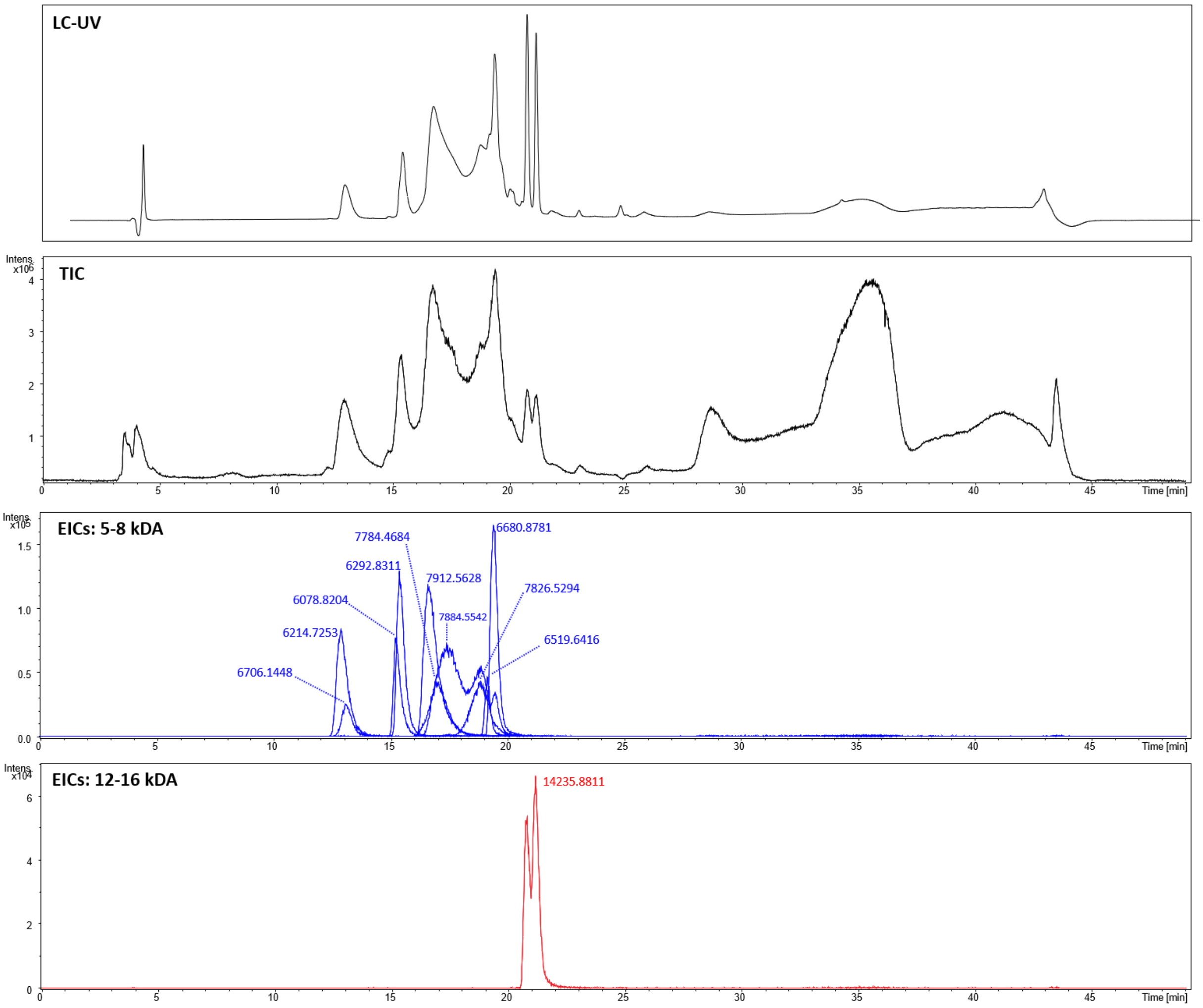

Supplementary Figure S17. LC-UV-MS chromatogram alignments highlight the abundant venom toxins in *Pseudonaja aspidorhyncha* (PAS22) venom. LC-UV peaks indicate the relative protein abundancy following separation. TIC shows the summary of all measured intensities. EICs display the extracted, high-abundant masses in the venom. EICs are sorted based on their mass range to enhance visibility (i.e., mass ranges of 5-8 kDa and 12-16 kDa). Key: LC-UV, liquid chromatography coupled to ultraviolet detection; TIC, Total Ion Chromatogram; EICs, Extracted Ion Chromatograms; MS, mass spectrometry.

Pseudonaja aspidorhyncha - Streaky Bay (PAS32)

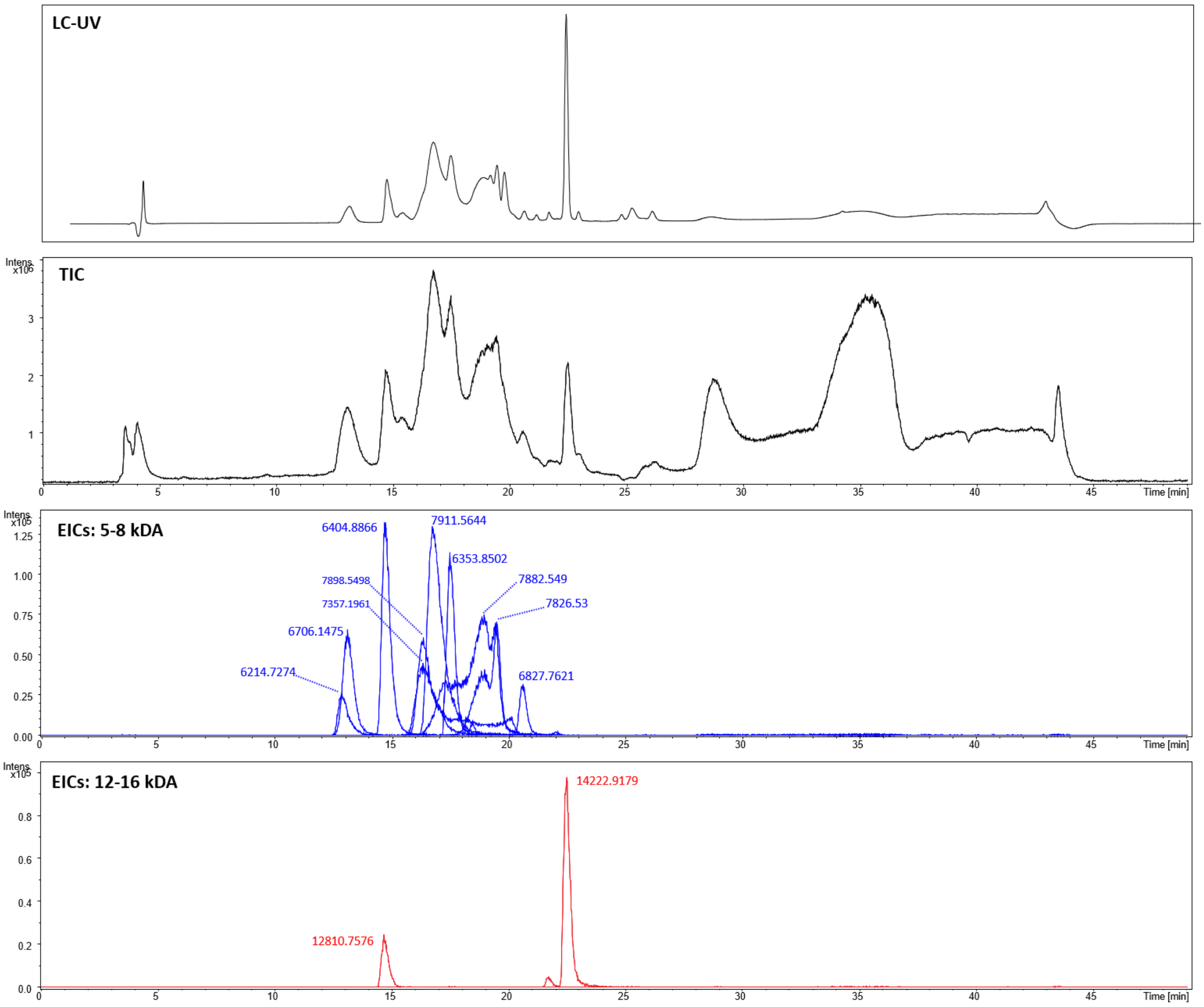

Supplementary Figure S18. LC-UV-MS chromatogram alignments highlight the abundant venom toxins in *Pseudonaja aspidorhyncha* (PAS32) venom. LC-UV peaks indicate the relative protein abundancy following separation. TIC shows the summary of all measured intensities. EICs display the extracted, high-abundant masses in the venom. EICs are sorted based on their mass range to enhance visibility (i.e., mass ranges of 5-8 kDa and 12-16 kDa). Key: LC-UV, liquid chromatography coupled to ultraviolet detection; TIC, Total Ion Chromatogram; EICs, Extracted Ion Chromatograms; MS, mass spectrometry.

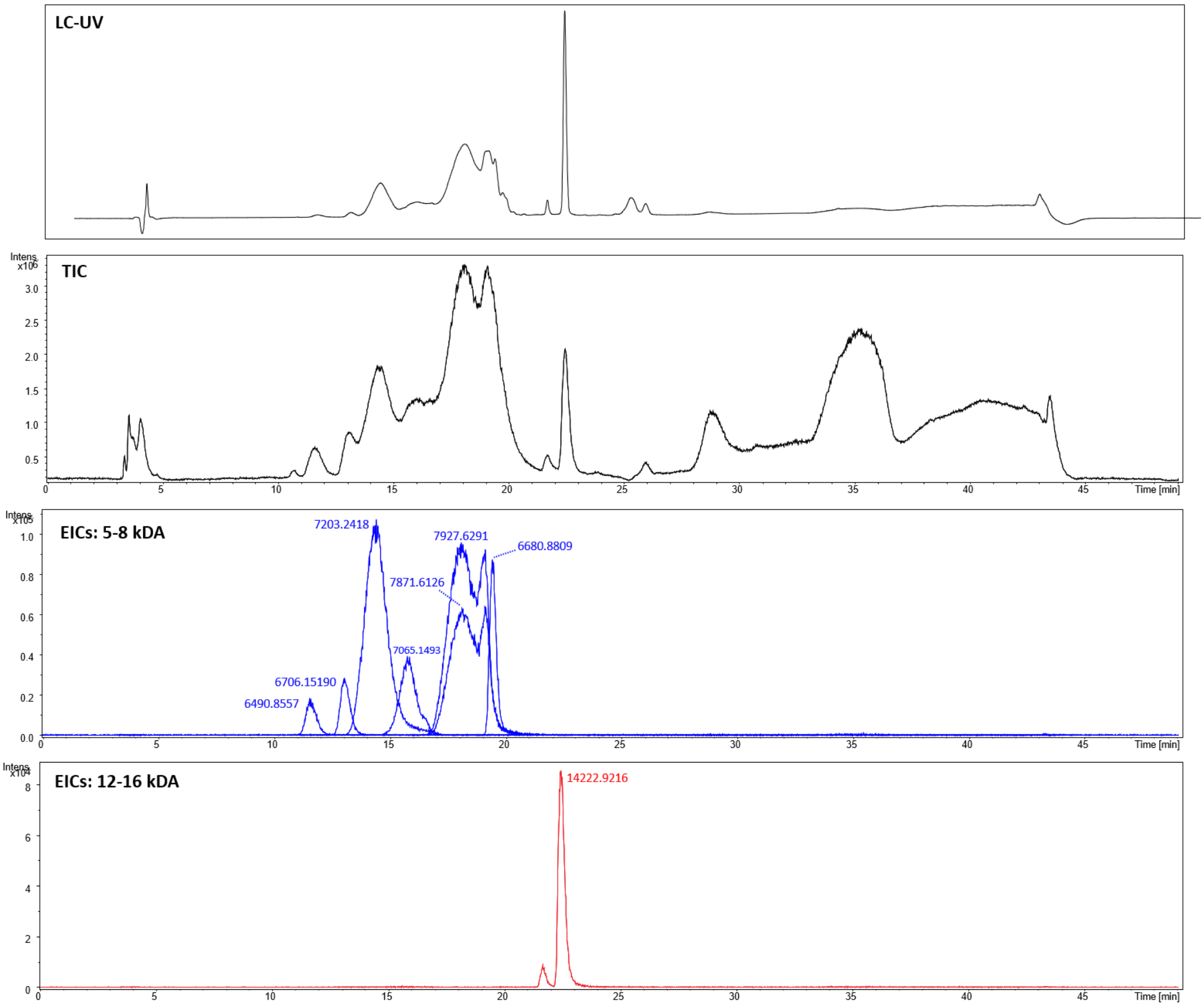

Supplementary Figure S19. LC-UV-MS chromatogram alignments highlight the abundant venom toxins in *Pseudonaja aspidorhyncha* (PAS25) venom. LC-UV peaks indicate the relative protein abundancy following separation. TIC shows the summary of all measured intensities. EICs display the extracted, high-abundant masses in the venom. EICs are sorted based on their mass range to enhance visibility (i.e., mass ranges of 5-8 kDa and 12-16 kDa). Key: LC-UV, liquid chromatography coupled to ultraviolet detection; TIC, Total Ion Chromatogram; EICs, Extracted Ion Chromatograms; MS, mass spectrometry.

Pseudonaja guttata - Longreach (SB22)

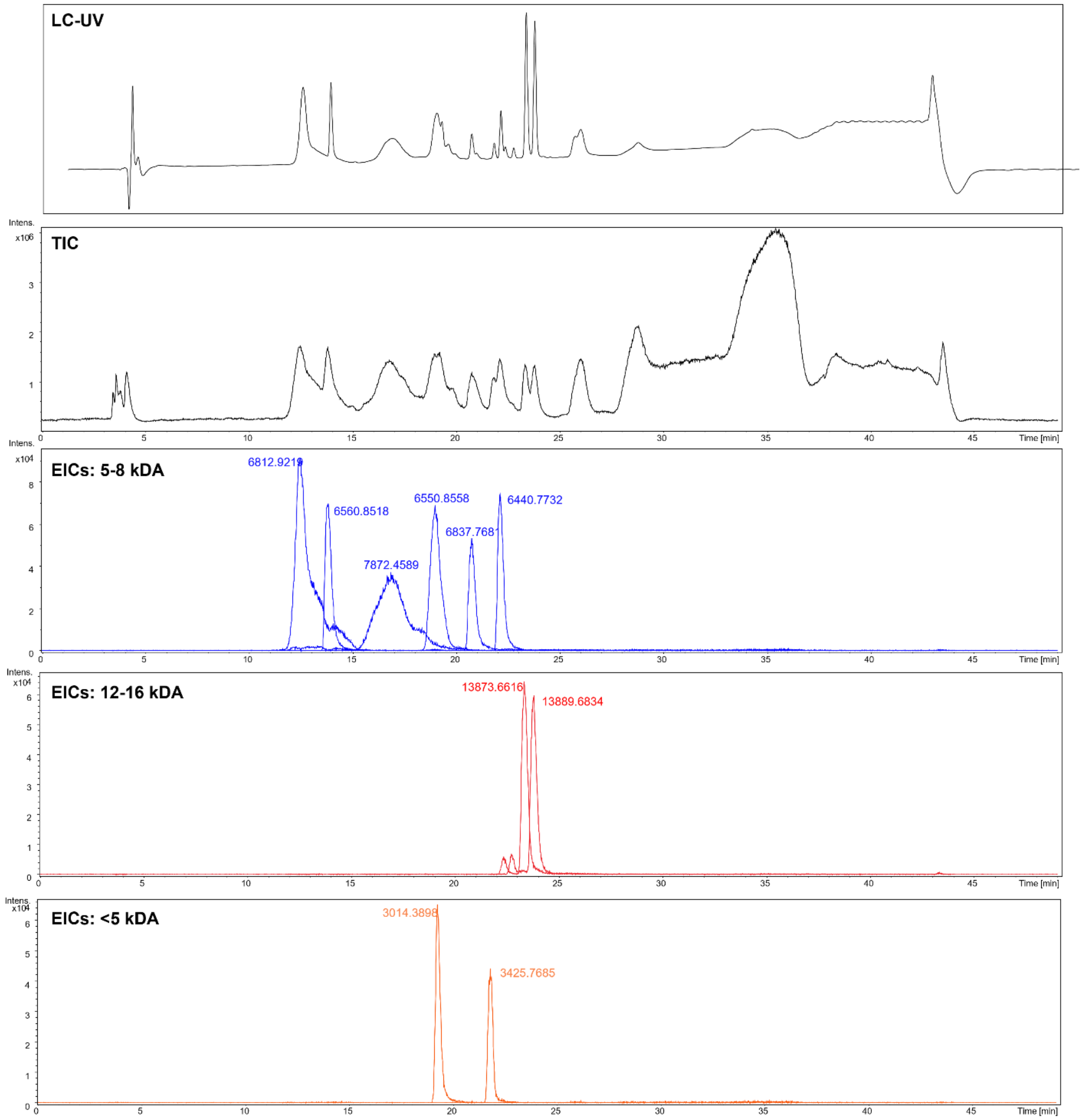

Supplementary Figure S20. LC-UV-MS chromatogram alignments highlight the abundant venom toxins in *Pseudonaja guttata* (SB22) venom. LC-UV peaks indicate the relative protein abundancy following separation. TIC shows the summary of all measured intensities. EICs display the extracted, high-abundant masses in the venom. EICs are sorted based on their mass range to enhance visibility (i.e., mass ranges of 5-8 kDa, 12-16 kDa and <5 kDa). Key: LC-UV, liquid chromatography coupled to ultraviolet detection; TIC, Total Ion Chromatogram; EICs, Extracted Ion Chromatograms; MS, mass spectrometry.

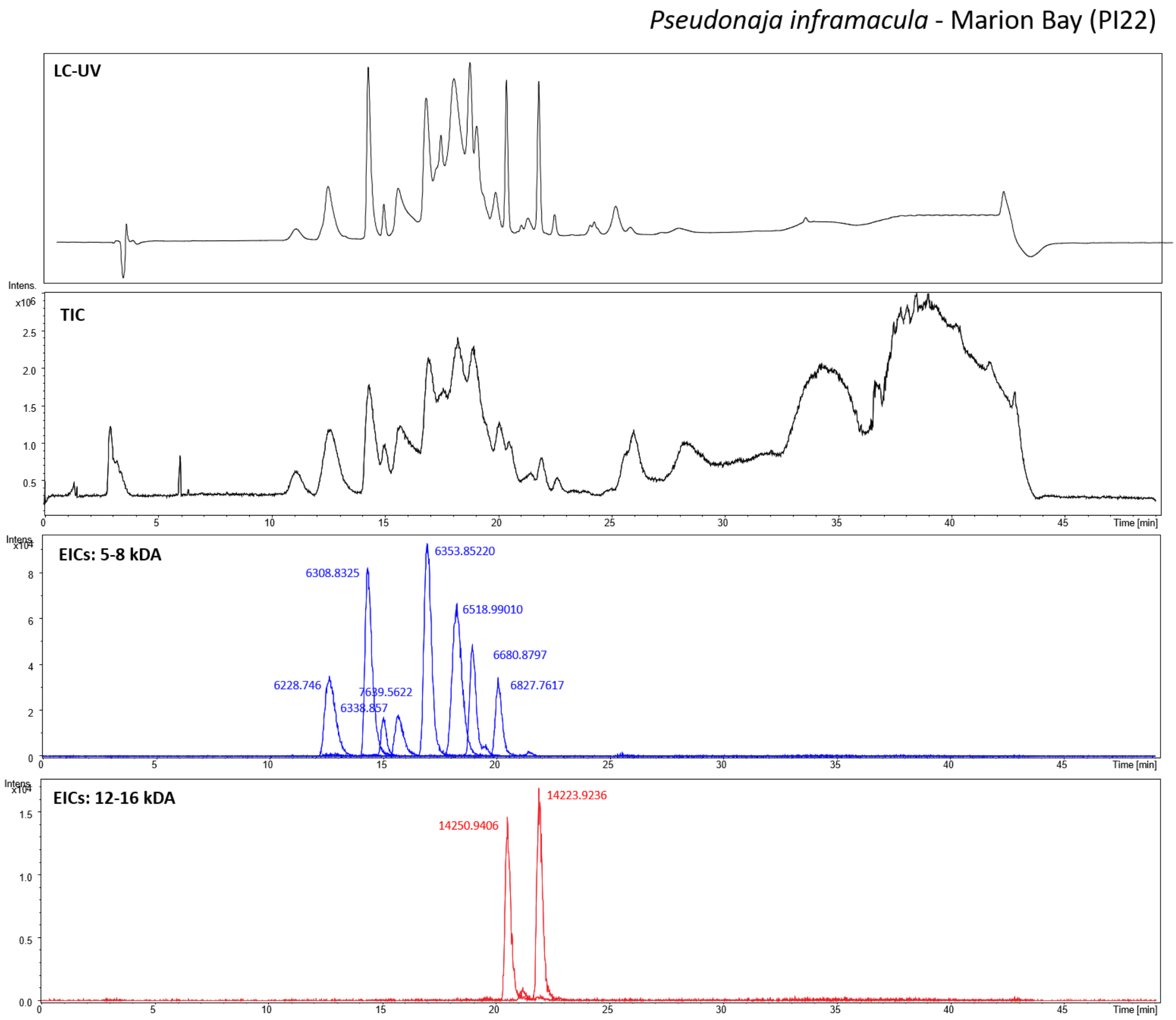

Supplementary Figure S21. LC-UV-MS chromatogram alignments highlight the abundant venom toxins in *Pseudonaja inframacula* (PI22) venom. LC-UV peaks indicate the relative protein abundancy following separation. TIC shows the summary of all measured intensities. EICs display the extracted, high-abundant masses in the venom. EICs are sorted based on their mass range to enhance visibility (i.e., mass ranges of 5-8 kDa and 12-16 kDa). Key: LC-UV, liquid chromatography coupled to ultraviolet detection; TIC, Total Ion Chromatogram; EICs, Extracted Ion Chromatograms; MS, mass spectrometry.

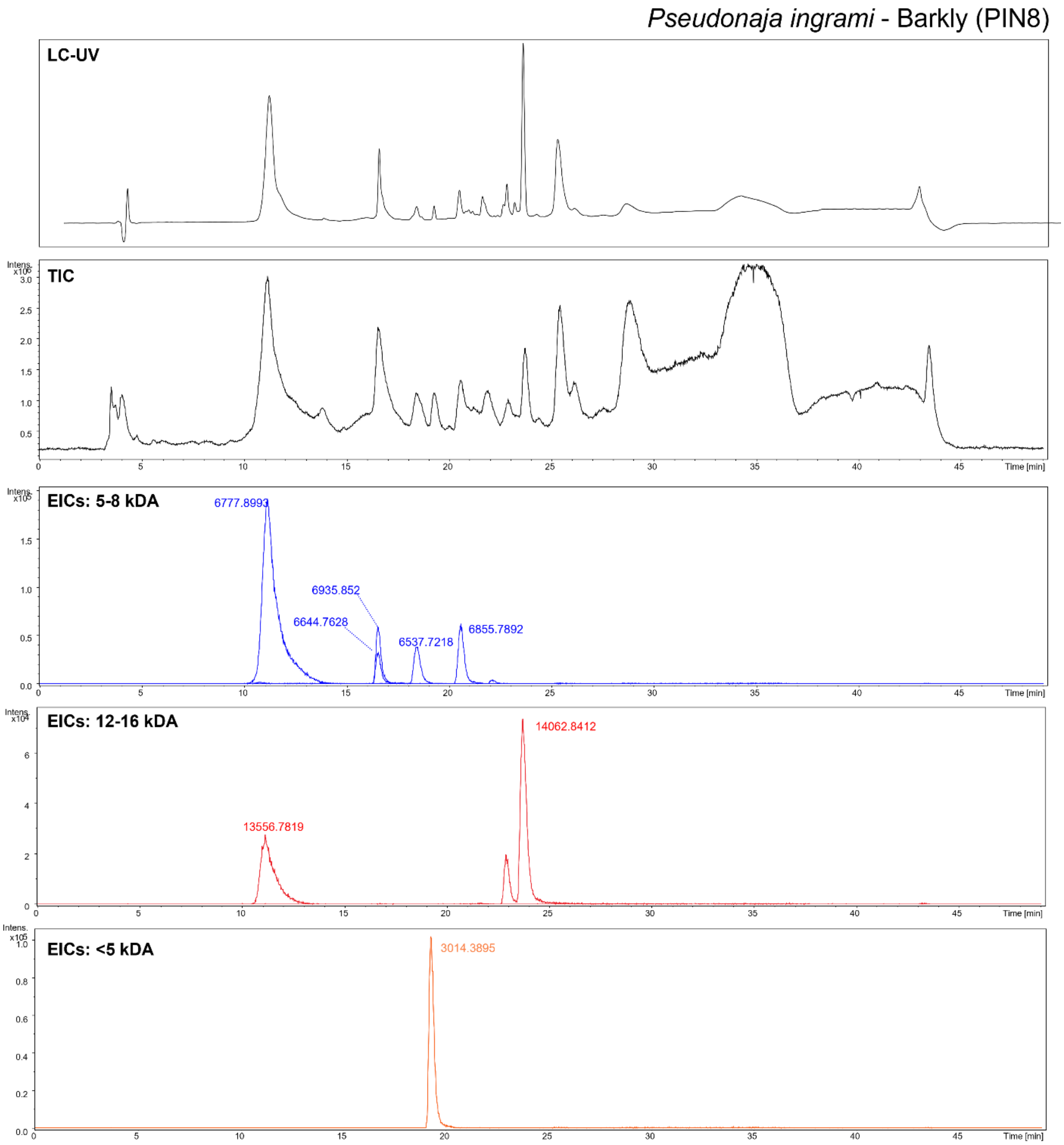

Supplementary Figure S22. LC-UV-MS chromatogram alignments highlight the abundant venom toxins in *Pseudonaja ingrami* (PIN8) venom. LC-UV peaks indicate the relative protein abundancy following separation. TIC shows the summary of all measured intensities. EICs display the extracted, high-abundant masses in the venom. EICs are sorted based on their mass range to enhance visibility (i.e., mass ranges of 5-8 kDa, 12-16 kDa and <5 kDa). Key: LC-UV, liquid chromatography coupled to ultraviolet detection; TIC, Total Ion Chromatogram; EICs, Extracted Ion Chromatograms; MS, mass spectrometry.

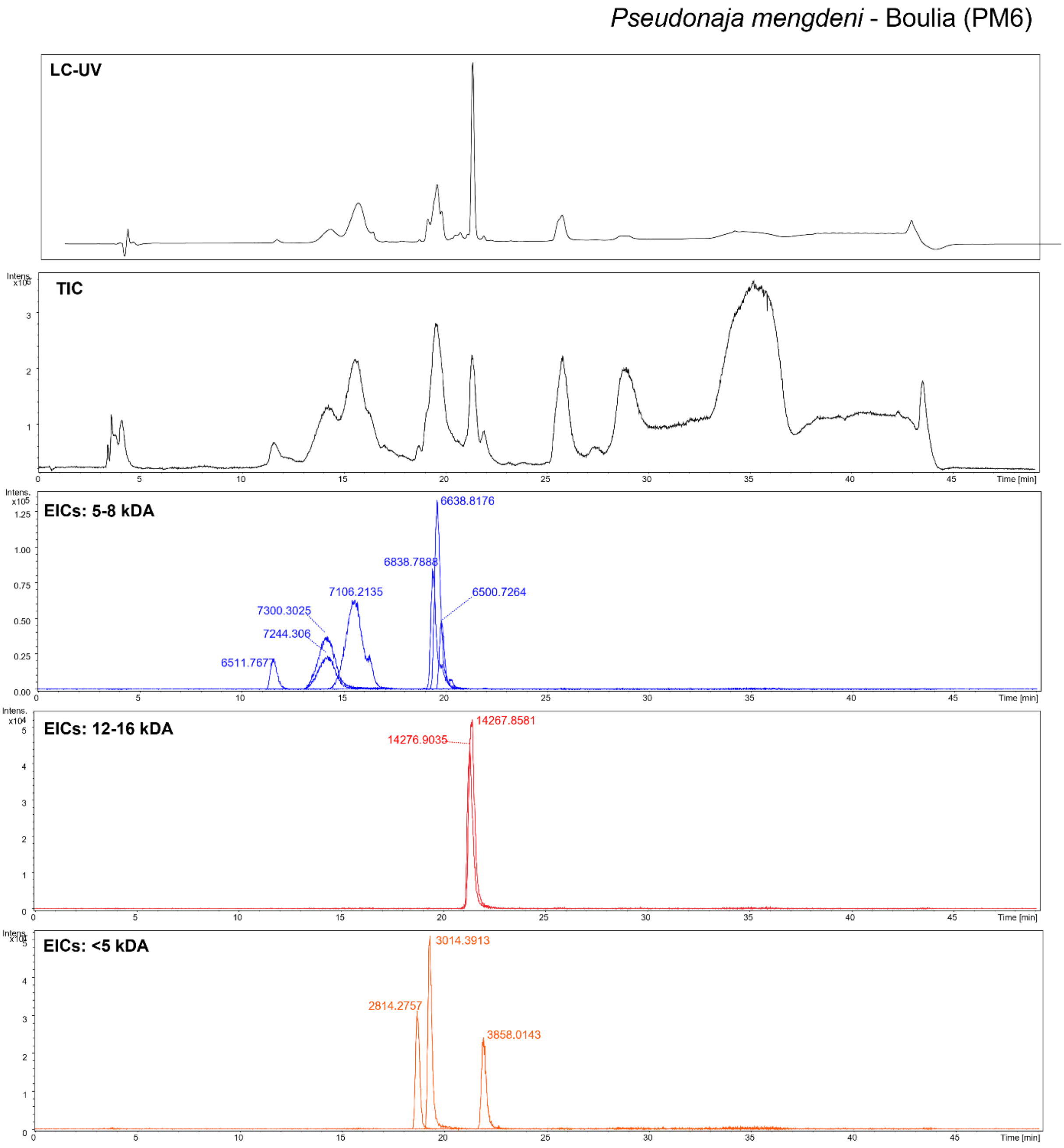

Supplementary Figure S23. LC-UV-MS chromatogram alignments highlight the abundant venom toxins in *Pseudonaja mengdeni* (PM6) venom. LC-UV peaks indicate the relative protein abundancy following separation. TIC shows the summary of all measured intensities. EICs display the extracted, high-abundant masses in the venom. EICs are sorted based on their mass range to enhance visibility (i.e., mass ranges of 5-8 kDa, 12-16 kDa and <5 kDa). Key: LC-UV, liquid chromatography coupled to ultraviolet detection; TIC, Total Ion Chromatogram; EICs, Extracted Ion Chromatograms; MS, mass spectrometry.

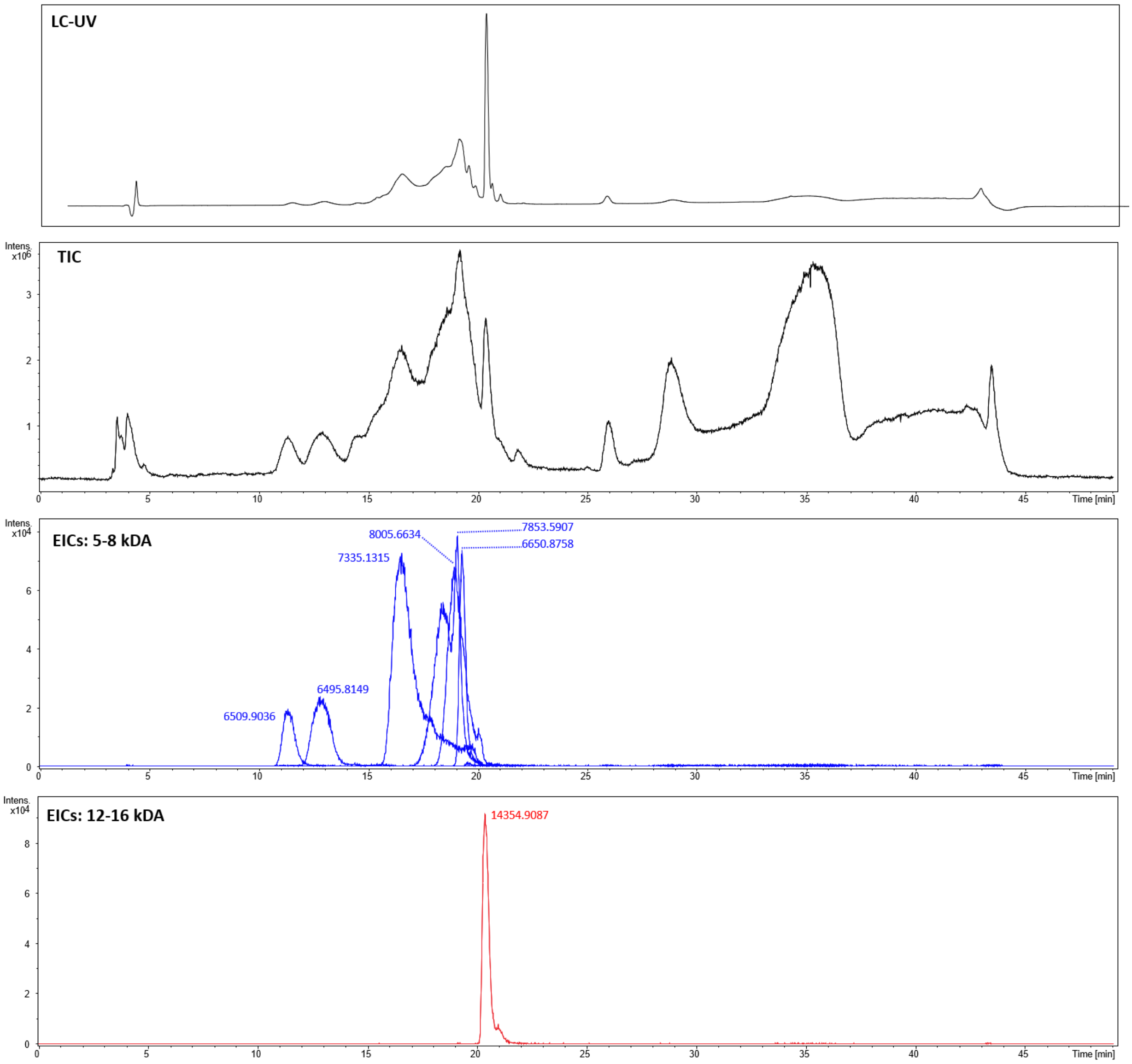

Supplementary Figure S24. LC-UV-MS chromatogram alignments highlight the abundant venom toxins in *Pseudonaja mengdeni* (PM8) venom. LC-UV peaks indicate the relative protein abundancy following separation. TIC shows the summary of all measured intensities. EICs display the extracted, high-abundant masses in the venom. EICs are sorted based on their mass range to enhance visibility (i.e., mass ranges of 5-8 kDa and 12-16 kDa). Key: LC-UV, liquid chromatography coupled to ultraviolet detection; TIC, Total Ion Chromatogram; EICs, Extracted Ion Chromatograms; MS, mass spectrometry.

Pseudonaja modesta - Carnarvon (PMOD13)

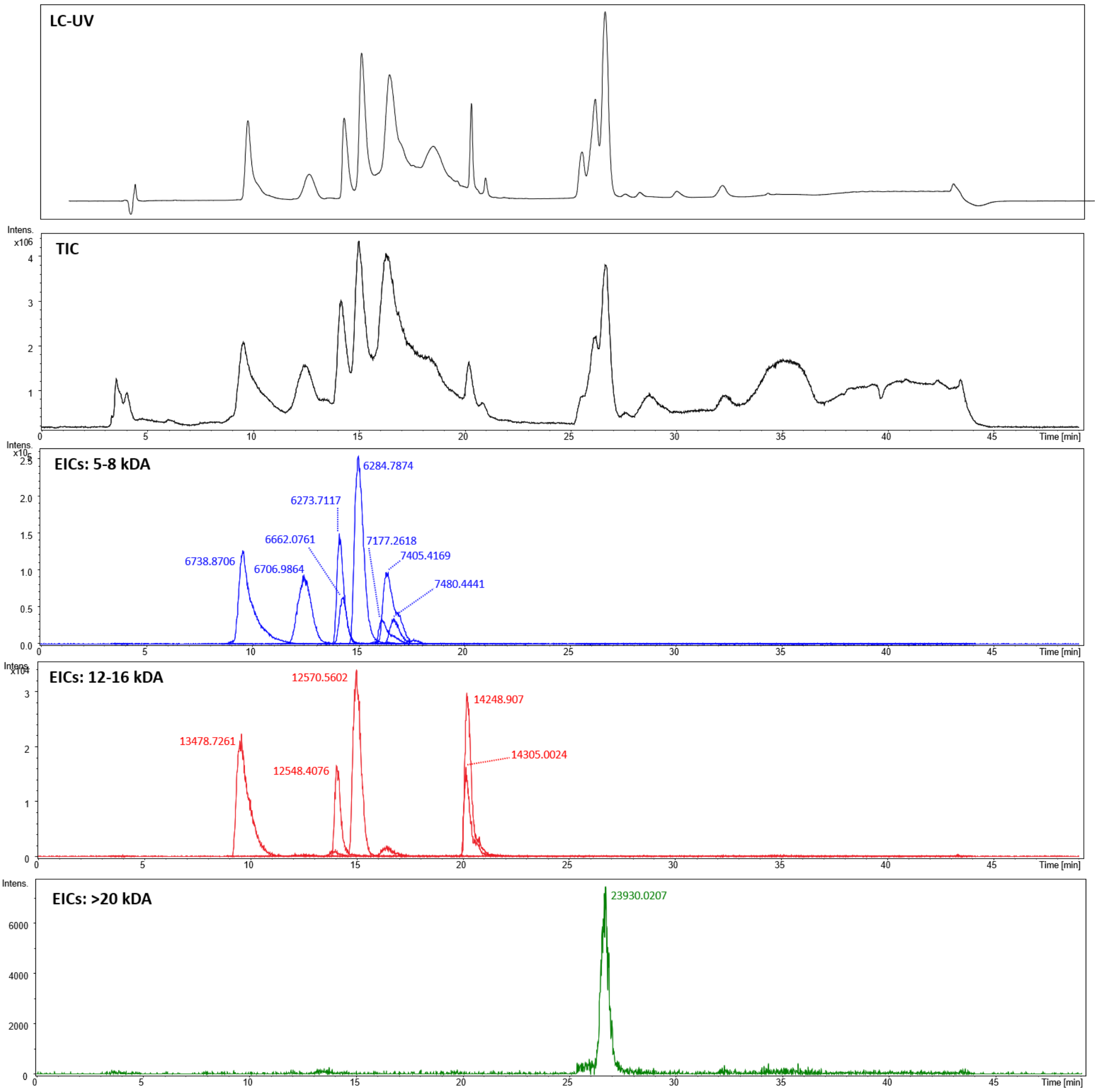

Supplementary Figure S25. LC-UV-MS chromatogram alignments highlight the abundant venom toxins in *Pseudonaja modesta* (PMOD13) venom. LC-UV peaks indicate the relative protein abundancy following separation. TIC shows the summary of all measured intensities. EICs display the extracted, high-abundant masses in the venom. EICs are sorted based on their mass range to enhance visibility (i.e., mass ranges of 5-8 kDa, 12-16 kDa and >20 kDa). Key: LC-UV, liquid chromatography coupled to ultraviolet detection; TIC, Total Ion Chromatogram; EICs, Extracted Ion Chromatograms; MS, mass spectrometry.

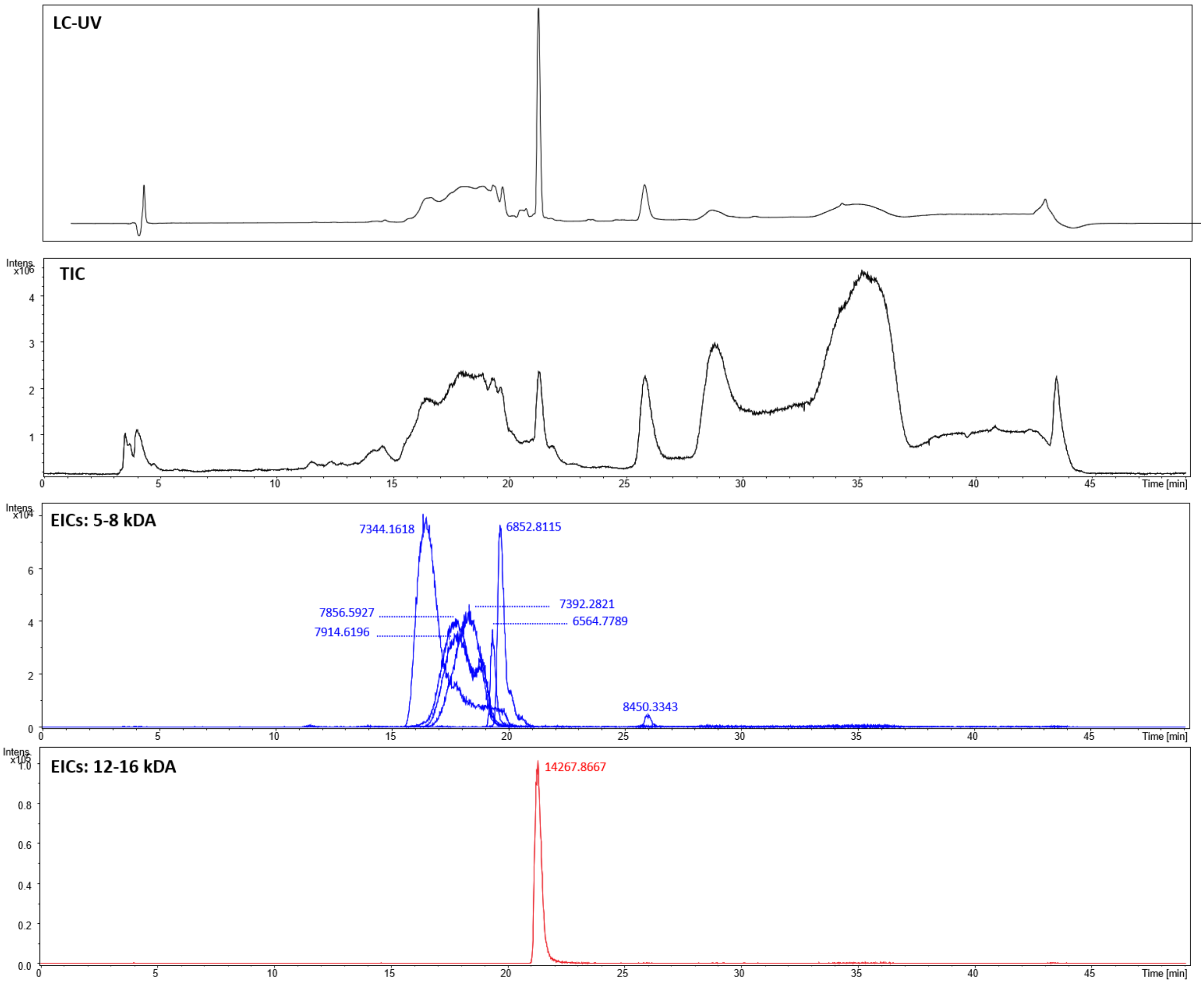

Supplementary Figure S26. LC-UV-MS chromatogram alignments highlight the abundant venom toxins in *Pseudonaja nuchalis* (PN37) venom. LC-UV peaks indicate the relative protein abundancy following separation. TIC shows the summary of all measured intensities. EICs display the extracted, high-abundant masses in the venom. EICs are sorted based on their mass range to enhance visibility (i.e., mass ranges of 5-8 kDa and 12-16 kDa). Key: LC-UV, liquid chromatography coupled to ultraviolet detection; TIC, Total Ion Chromatogram; EICs, Extracted Ion Chromatograms; MS, mass spectrometry.

Pseudonaja nuchalis - Tennant Creek (PN38)

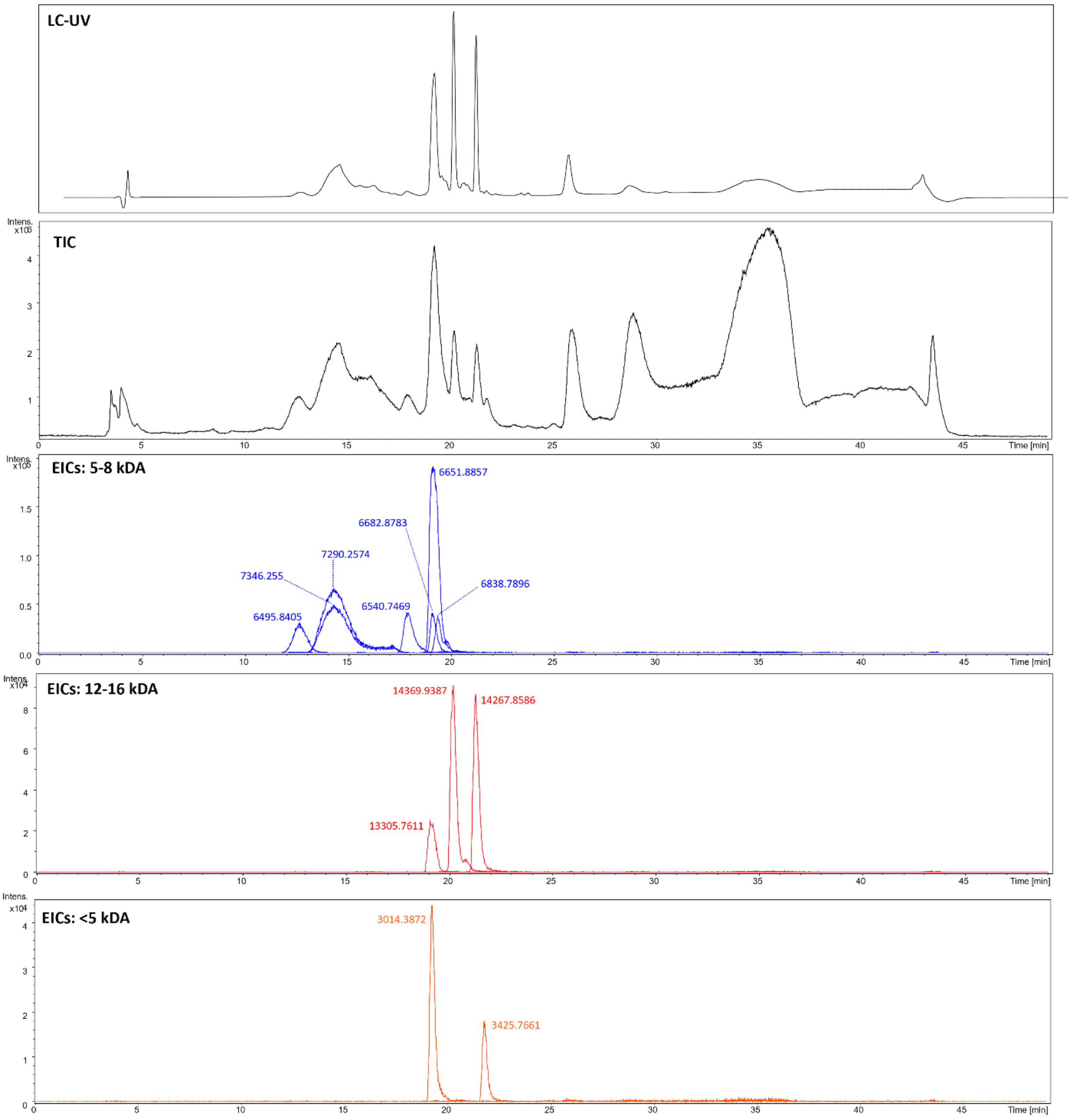

Supplementary Figure S27. LC-UV-MS chromatogram alignments highlight the abundant venom toxins in *Pseudonaja nuchalis* (PN38) venom. LC-UV peaks indicate the relative protein abundancy following separation. TIC shows the summary of all measured intensities. EICs display the extracted, high-abundant masses in the venom. EICs are sorted based on their mass range to enhance visibility (i.e., mass ranges of 5-8 kDa, 12-16 kDa and <5 kDa). Key: LC-UV, liquid chromatography coupled to ultraviolet detection; TIC, Total Ion Chromatogram; EICs, Extracted Ion Chromatograms; MS, mass spectrometry.

Pseudonaja textilis - Alice Springs (CB493)

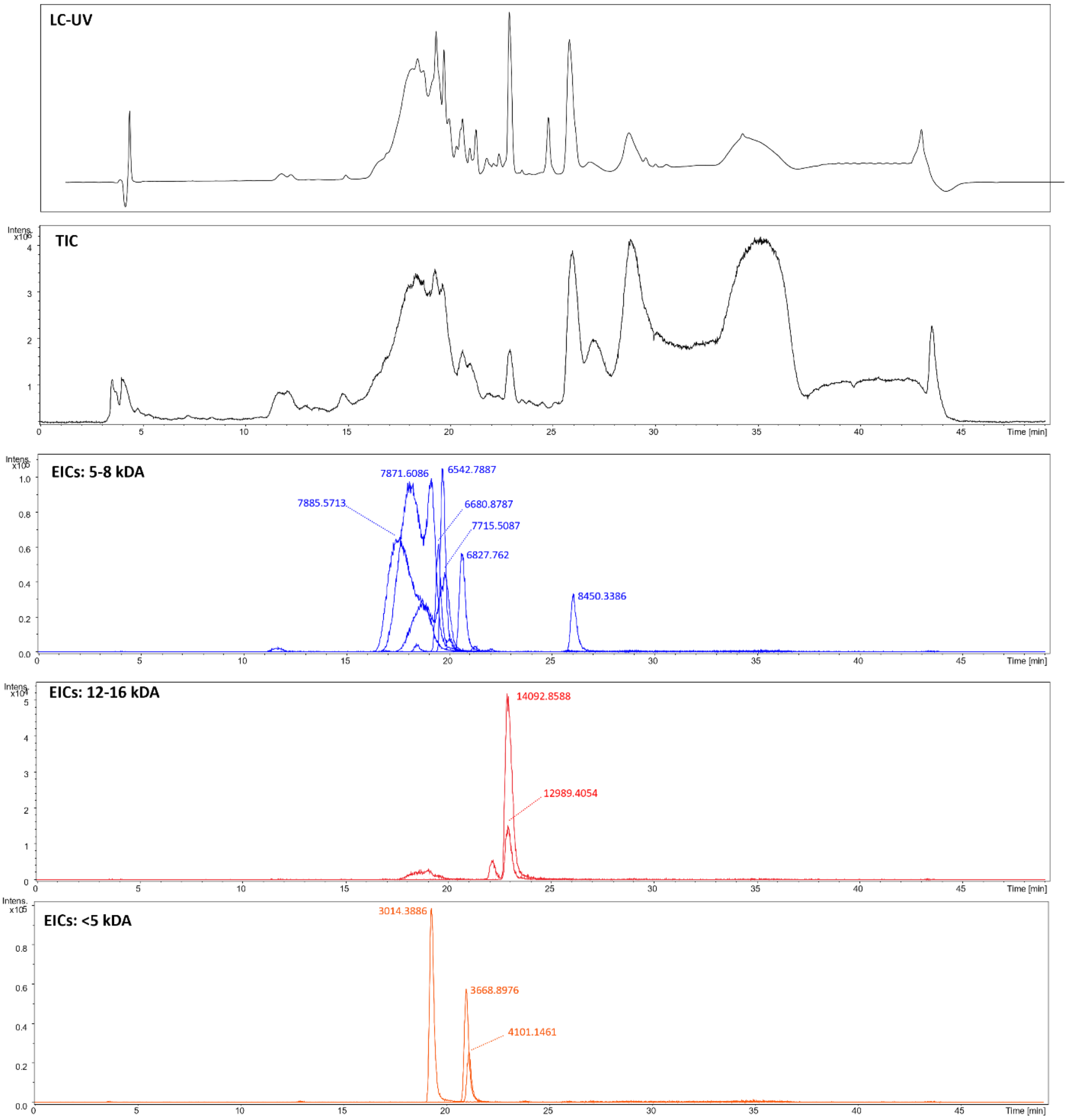

Supplementary Figure S28. LC-UV-MS chromatogram alignments highlight the abundant venom toxins in *Pseudonaja textilis* (CB493) venom. LC-UV peaks indicate the relative protein abundancy following separation. TIC shows the summary of all measured intensities. EICs display the extracted, high-abundant masses in the venom. EICs are sorted based on their mass range to enhance visibility (i.e., mass ranges of 5-8 kDa, 12-16 kDa and <5 kDa). Key: LC-UV, liquid chromatography coupled to ultraviolet detection; TIC, Total Ion Chromatogram; EICs, Extracted Ion Chromatograms; MS, mass spectrometry.

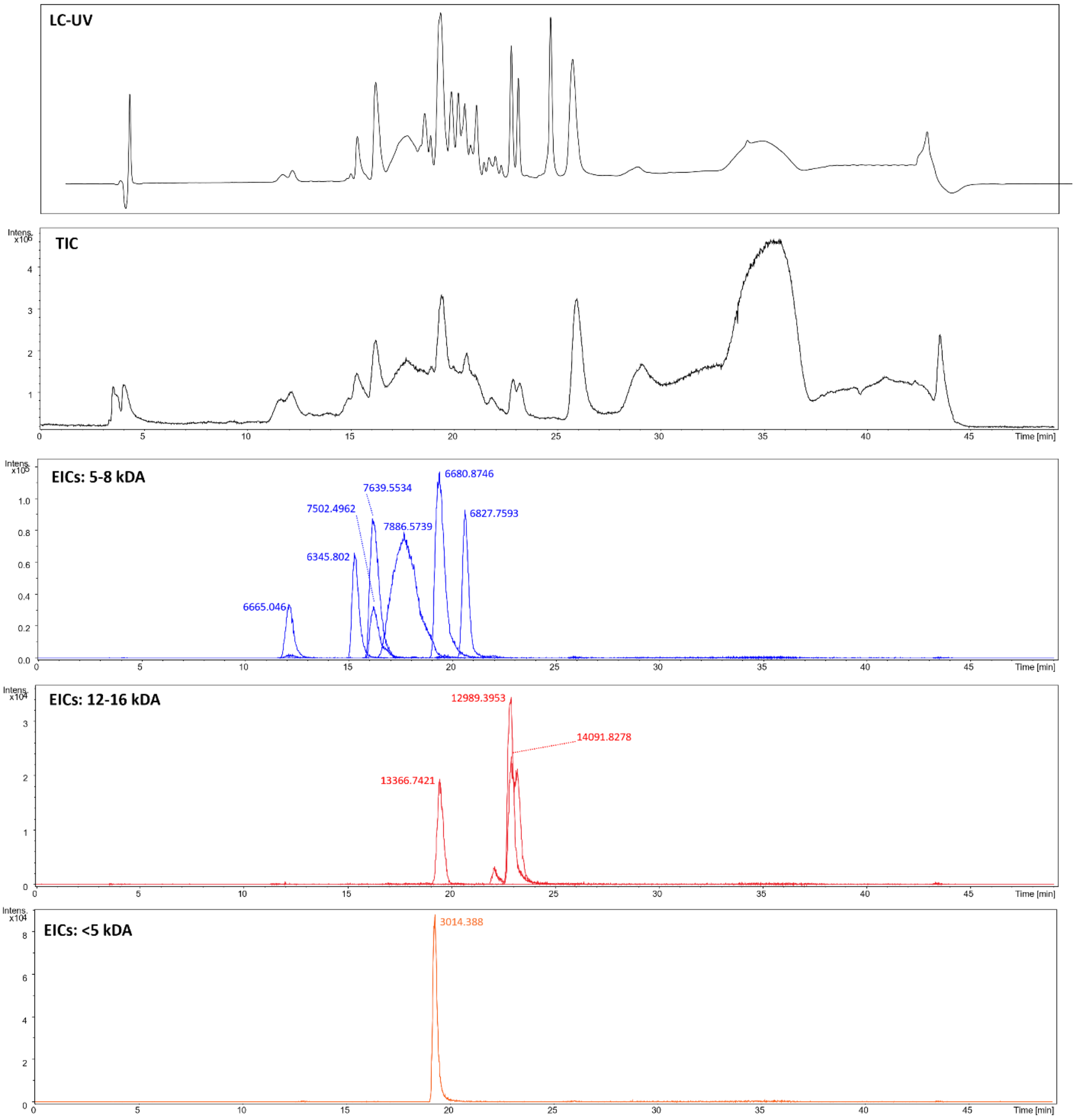

Supplementary Figure S29. LC-UV-MS chromatogram alignments highlight the abundant venom toxins in *Pseudonaja textilis* (CB479) venom. LC-UV peaks indicate the relative protein abundancy following separation. TIC shows the summary of all measured intensities. EICs display the extracted, high-abundant masses in the venom. EICs are sorted based on their mass range to enhance visibility (i.e., mass ranges of 5-8 kDa, 12-16 kDa and <5 kDa). Key: LC-UV, liquid chromatography coupled to ultraviolet detection; TIC, Total Ion Chromatogram; EICs, Extracted Ion Chromatograms; MS, mass spectrometry.

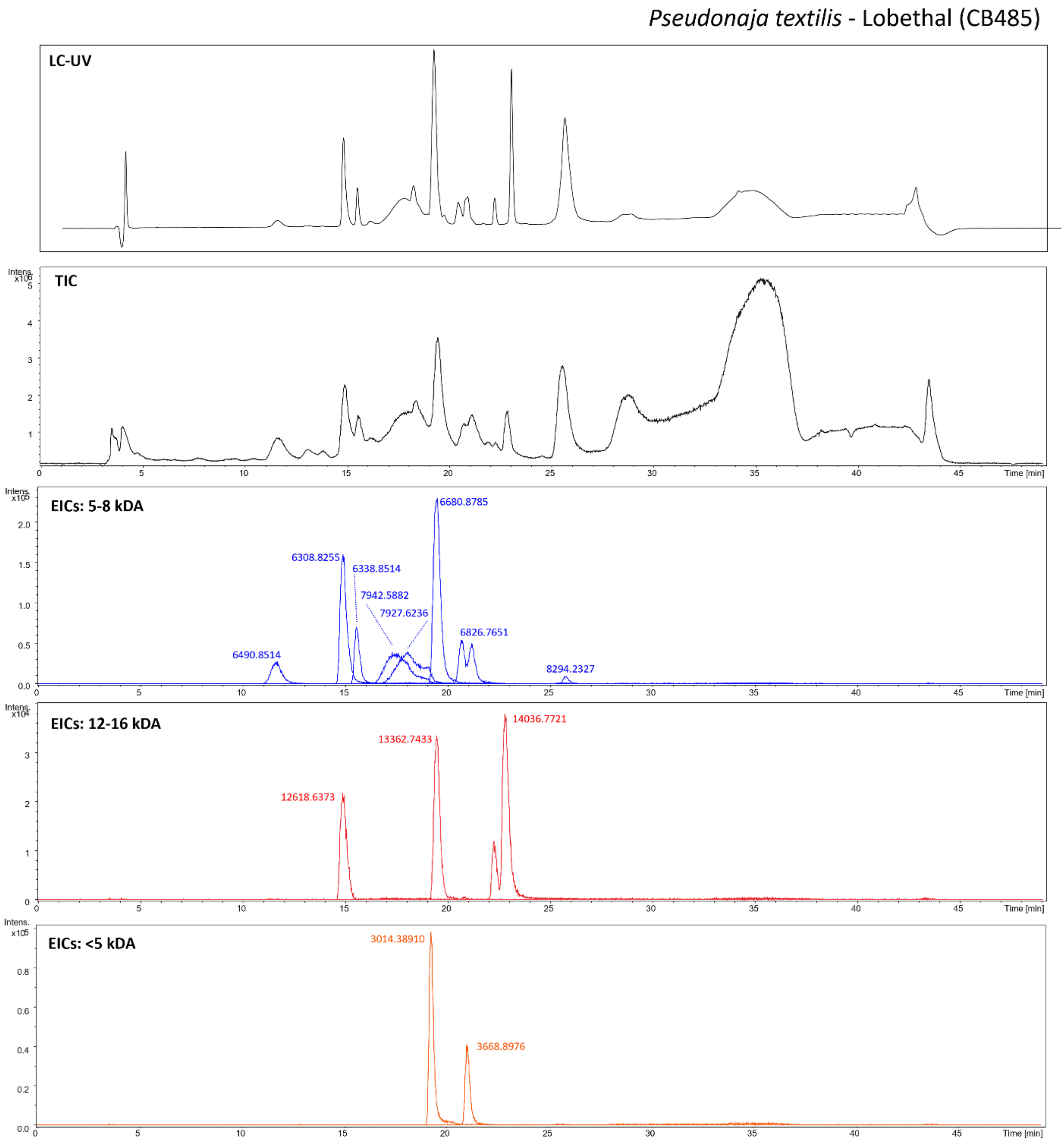

Supplementary Figure S30. LC-UV-MS chromatogram alignments highlight the abundant venom toxins in *Pseudonaja textilis* (CB485) venom. LC-UV peaks indicate the relative protein abundancy following separation. TIC shows the summary of all measured intensities. EICs display the extracted, high-abundant masses in the venom. EICs are sorted based on their mass range to enhance visibility (i.e., mass ranges of 5-8 kDa, 12-16 kDa and <5 kDa). Key: LC-UV, liquid chromatography coupled to ultraviolet detection; TIC, Total Ion Chromatogram; EICs, Extracted Ion Chromatograms; MS, mass spectrometry.

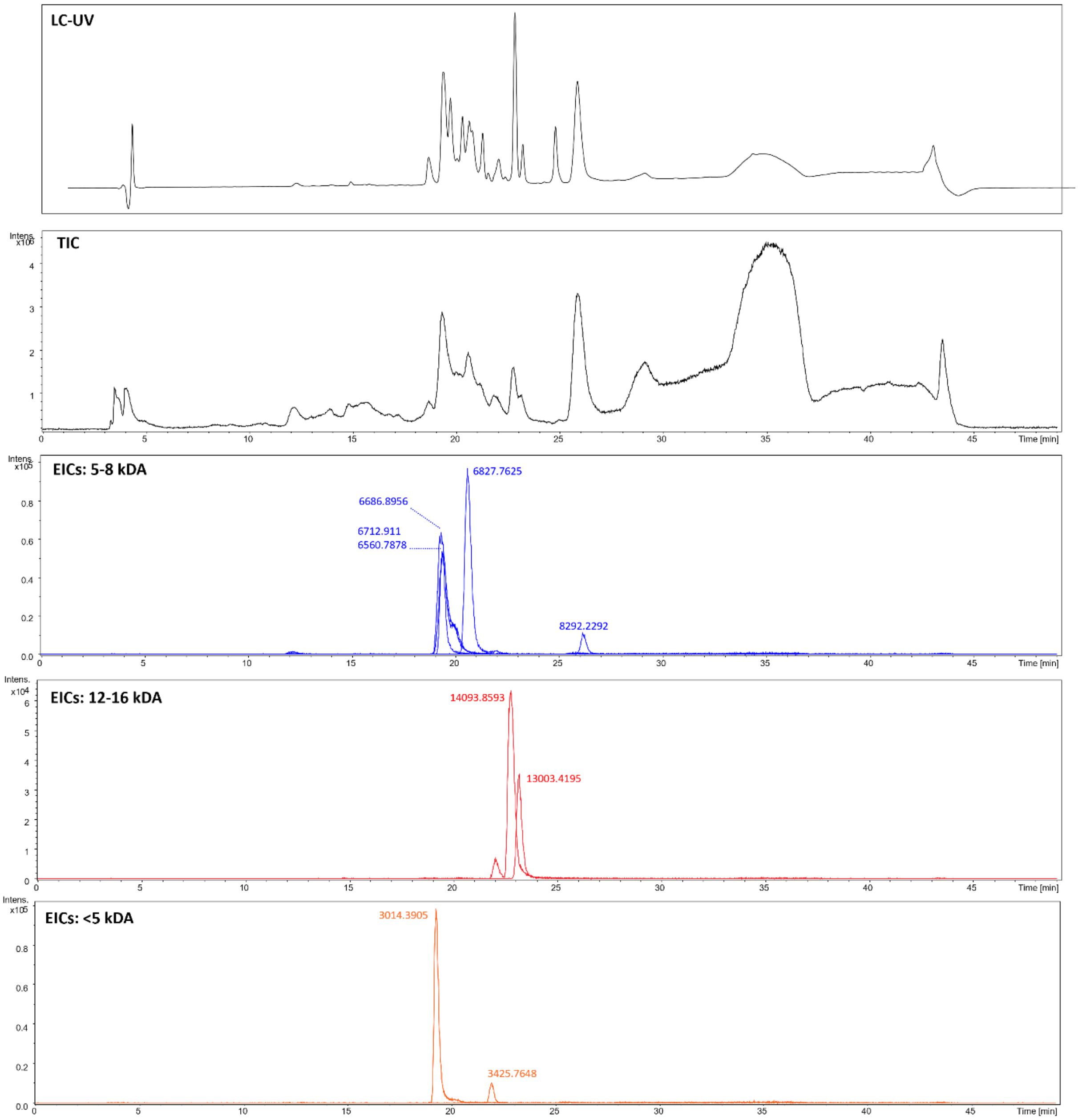

Supplementary Figure S31. LC-UV-MS chromatogram alignments highlight the abundant venom toxins in *Pseudonaja textilis* (CB467) venom. LC-UV peaks indicate the relative protein abundancy following separation. TIC shows the summary of all measured intensities. EICs display the extracted, high-abundant masses in the venom. EICs are sorted based on their mass range to enhance visibility (i.e., mass ranges of 5-8 kDa, 12-16 kDa and <5 kDa). Key: LC-UV, liquid chromatography coupled to ultraviolet detection; TIC, Total Ion Chromatogram; EICs, Extracted Ion Chromatograms; MS, mass spectrometry.

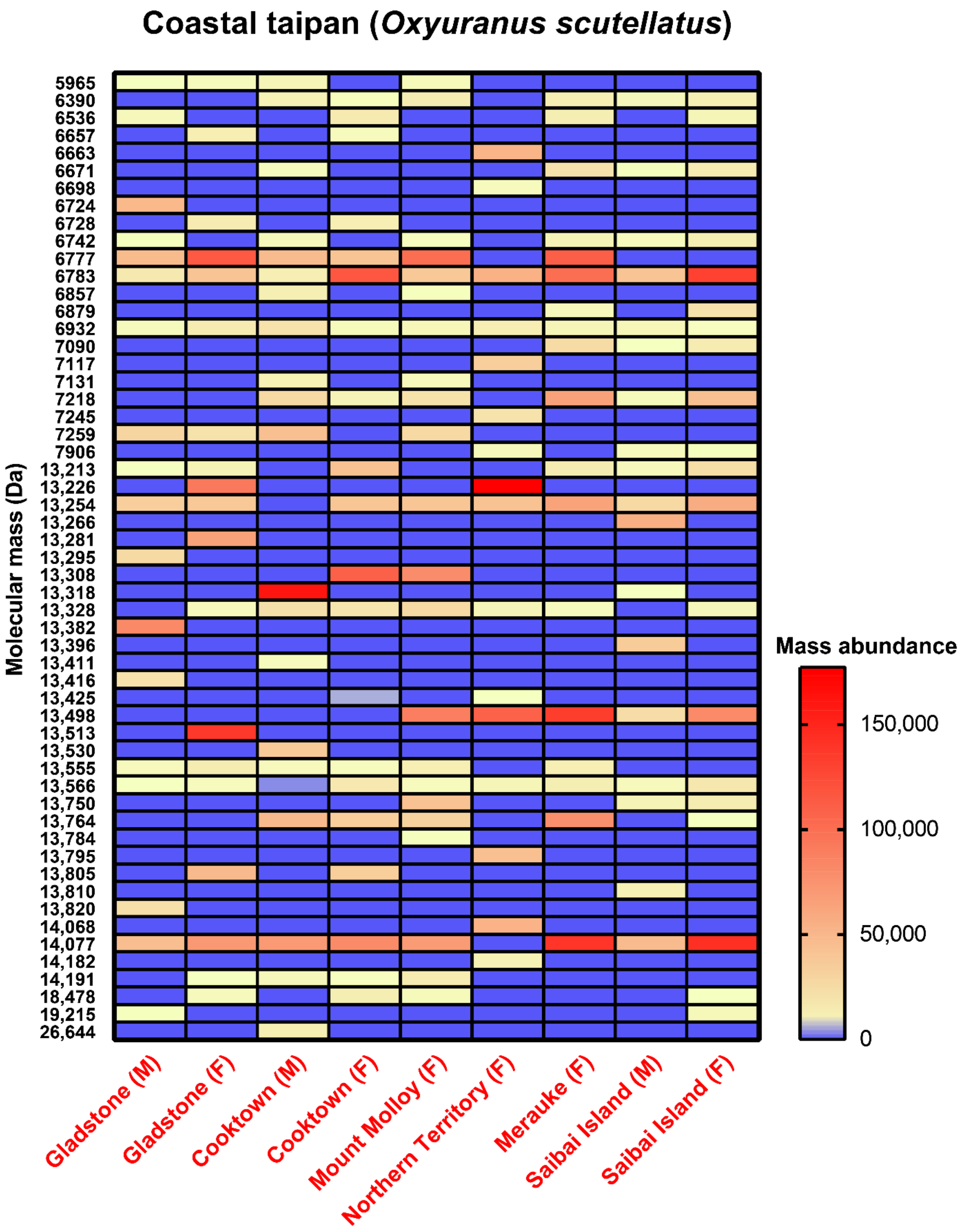

Supplementary Figure S32. Heatmap visualization of comparative protein abundances across coastal taipan (*Oxyuranus scutellatus*) venoms. Red or yellow indicate the presence of a mass and blue indicates the absence of a mass. The mass abundance is based on the measured intensity in each venom. Key: Da, Dalton; M, Male; F, Female.

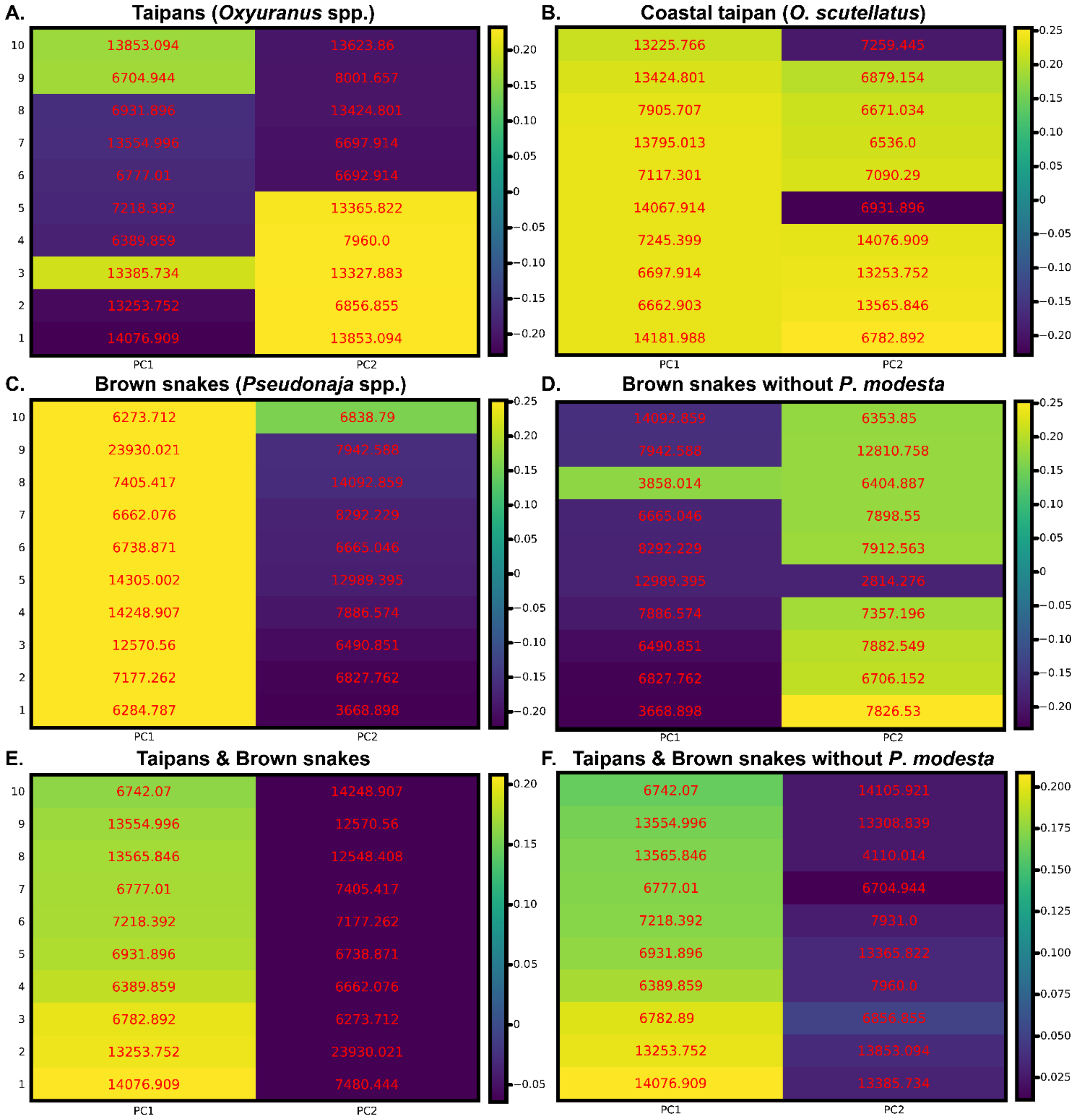

Supplementary Figure S33. Principal component analysis (PCA) loading scores of taipan (*Oxyuranus* spp.) and brown snake (*Pseudonaja* spp.) venoms. The 10 venom protein masses (Da) with the highest loading scores for PC1 and PC2 are shown. The color gradient ranges from yellow to dark blue, with yellow representing positive values and dark blue representing negative values. The higher the absolute value of the loading, the more relevant that variable becomes to define the PC. (**A**) Loading plots of PCA displayed in Figure 4A. (**B**) Loading plots of PCA displayed in Figure 4B. (**C**) Loading plots of PCA displayed in Figure 4C. (**D**) Loading plots of PCA displayed in Figure 4D. (**E**) Loading plots of PCA displayed in Figure 4E. (**F**) Loading plots of PCA displayed in Figure 4F.
